# CETSA screening identifies known and novel thymidylate synthase inhibitors and slow intracellular activation of 5-fluorouracil

**DOI:** 10.1038/ncomms11040

**Published:** 2016-03-24

**Authors:** Helena Almqvist, Hanna Axelsson, Rozbeh Jafari, Chen Dan, André Mateus, Martin Haraldsson, Andreas Larsson, Daniel Martinez Molina, Per Artursson, Thomas Lundbäck, Pär Nordlund

**Affiliations:** 1 Laboratories for Chemical Biology, Karolinska Institutet, Science for Life Laboratory Stockholm, Division of Translational Medicine & Chemical Biology, Department of Medical Biochemistry & Biophysics, Karolinska Institutet, Tomtebodavägen 23A, Solna 171 65, Sweden; 2 Department of Medical Biochemistry & Biophysics, Division of Biophysics, Karolinska Institutet, Scheeles väg 2, Stockholm 171 77, Sweden; 3 School of Biological Sciences, Nanyang Technological University, 61 Biopolis Drive (Proteos), Singapore 138673, Singapore; 4 Department of Pharmacy, Uppsala University, BMC, Box 580, Uppsala SE-751 23, Sweden; 5 School of Biological Sciences, Nanyang Technological University, SBS-04s-45, 60 Nanyang Drive, Singapore 639798, Singapore; 6 Uppsala University Drug Optimization and Pharmaceutical Profiling Platform (UDOPP), Department of Pharmacy, Uppsala University, BMC, Box 580, Uppsala SE-751 23, Sweden; 7 Science for Life Laboratory Drug Discovery and Development platform, Uppsala University, Uppsala SE-751 23, Sweden; 8 Institute of Cellular and Molecular Biology, ASTAR, 61 Biopolis Drive (Proteos), Singapore 138673, Singapore

## Abstract

Target engagement is a critical factor for therapeutic efficacy. Assessment of compound binding to native target proteins in live cells is therefore highly desirable in all stages of drug discovery. We report here the first compound library screen based on biophysical measurements of intracellular target binding, exemplified by human thymidylate synthase (TS). The screen selected accurately for all the tested known drugs acting on TS. We also identified TS inhibitors with novel chemistry and marketed drugs that were not previously known to target TS, including the DNA methyltransferase inhibitor decitabine. By following the cellular uptake and enzymatic conversion of known drugs we correlated the appearance of active metabolites over time with intracellular target engagement. These data distinguished a much slower activation of 5-fluorouracil when compared with nucleoside-based drugs. The approach establishes efficient means to associate drug uptake and activation with target binding during drug discovery.

## Abstract (web summary)

Drugs therapeutic efficacy relies on their capability of binding the relevant targets in a physiological environment, which has so far been hard to measure. Here, the authors present a compound library screen based on a target engagement assay that reports on protein stability upon ligands binding in cell.

## Graphical abstract

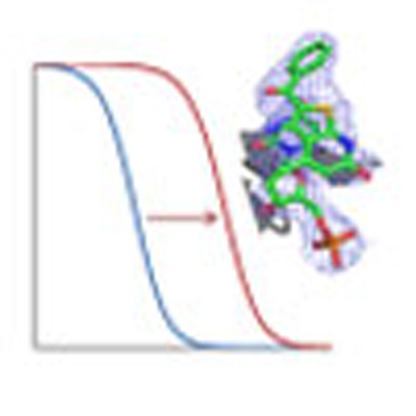

Therapeutic efficacy is achieved when drugs bind their relevant molecular targets in the physiologically relevant setting. Despite this known fact, insufficient control of target engagement is surprisingly common and contributes to high failure rates in clinical trials^1^,^2^,^3^. Methods that allow for robust measurements of drug target engagement in primary cells, tissues and patient biopsies are thus urgently needed, but have been hard to establish^4^,^5^.

Ligand-induced changes in protein thermal stability are frequently used to monitor binding to isolated proteins in thermal shift assays^6^,^7^,^8^,^9^. The recently developed cellular thermal shift assay (CETSA; see Supplementary Note 1 for a list of abbreviations) builds on the discovery that ligand induced thermal shifts can also be measured in the context of cell lysates, whole cells or tissues^10^. This finding effectively allows for biophysical binding studies in native environments—preserving expression levels, posttranslational modifications and the local environment for the endogenous protein. Whereas the original CETSA study included multiple case studies, recent work extends this method to include melting transitions for a significant portion of the proteome, thus expanding the putative use of the methodology to a large number of protein families^11^,^12^,^13^. Of practical importance is that the melting transitions are established for individual proteins by the use of protein affinity reagents^10^,^14^ or quantitative mass spectrometry (MS)^11^,^12^,^13^. As a consequence these measurements are amenable to either high-throughput measurements or proteome-wide multiplexing.

To improve current strategies for drug development, stringent control of target engagement should ideally be established from initial hit identification, through preclinical and clinical development. The same demands apply to the validation of chemical probes discovered in academic settings^2^,^4^,^15^. To probe the value of CETSA in earlier stages of the discovery process we applied it for primary screening of thymidylate synthase (TS) in live human myelogenous leukemia cells. TS is a pivotal enzyme in production of thymidine monophosphate and a well validated cancer target^16^,^17^. Inhibition of TS leads to thymineless death characterized by DNA-damage, chromosomal fragmentation and concomitant induction of apoptosis. Novel classes of TS inhibitors with improved efficacy and resistance profiles could provide important complements to current TS directed drugs, for which there are reports of resistance^18^,^19^.

Here, we show for the first time that a CETSA-based screen for direct physical target engagement constitutes an attractive high throughput screening (HTS) strategy, which allows for the detection of known and novel TS inhibitors with cellular activity. Furthermore, we establish a hit validation strategy, in which time-dependent target engagement is explored in parallel with measurement of intracellular compound concentration. Taken together this provides a sound and efficient strategy to establish control of target engagement from an early stage of the drug discovery process, and which is likely to minimize problems in subsequent stages.

## Results

### Microplate-based CETSA measuring target engagement of TS

**Figure 1:**
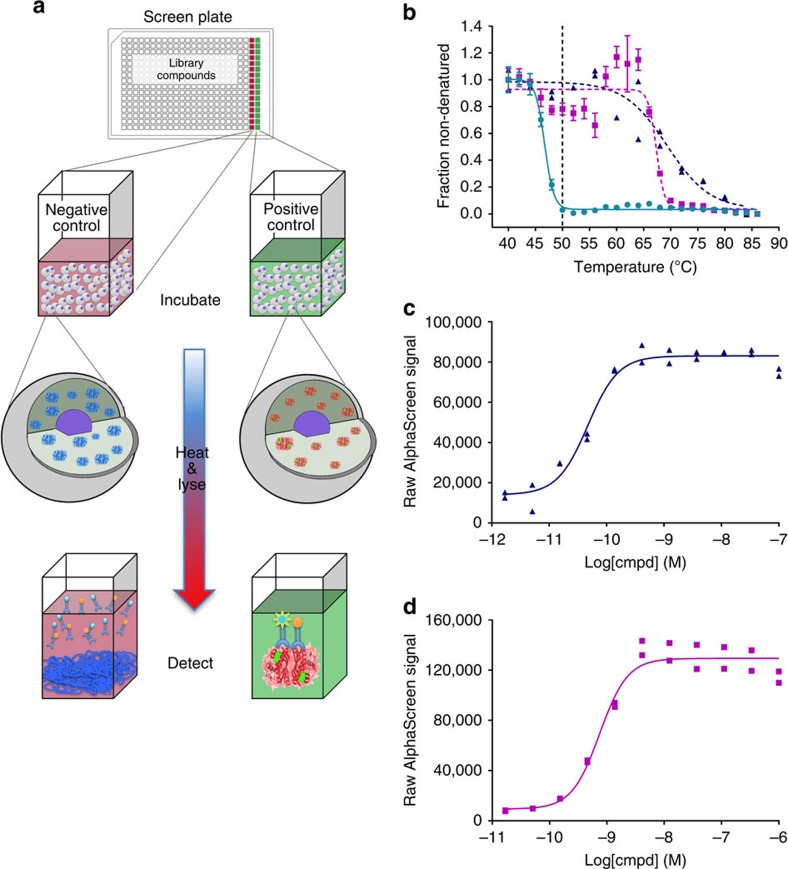
Development of a no-wash CETSA for human TS. (**a**) Overview of the assay principle with live K562 cells seeded into a 384-well PCR plate. The plate contains controls or library compounds that are taken up by the cells. Following a pre-incubation period the plate is transiently heated for 3 min followed by cooling and cell lysis. Part of the cell lysate is transferred to a detection plate, to which antibodies and AlphaScreen beads are added to allow measurements of remaining soluble TS. (**b**) CETSA derived *T*_agg_ curves for TS in K562 cells in the presence of DMSO (0.5%) (green circle), 15 μM floxuridine (blue triangle) or 1 μM raltitrexed (magenta square). All data were normalized to the response observed for each treatment condition at the lowest test temperature. The solid line represents the best fit to the Boltzmann sigmoid equation resulting in an apparent *T*_agg_ of 46.7±0.2 °C for the DMSO control, whereas both floxuridine and raltitrexed stabilized TS above 65 °C (we do not consider higher *T*_agg_ values reliable as these temperatures influence cell membrane integrity^10^). The vertical dotted line is at 50 °C, the temperature selected for the isothermal screen. Data are provided as the average and standard error of mean (s.e.m.) from two independent experiments performed in duplicate for raltitrexed and as individual data points from one experiment in duplicate for floxuridine. (**c**) ITDRF_CETSA_ of floxuridine (blue triangle) at 50 °C based on raw data from the AlphaScreen readings. The solid line represents the best fit to a saturation binding curve resulting in an EC_50_ of 47±16 pM. Data are provided as two individual data points from one test occasion. (**d**) The corresponding ITDRF_CETSA_ for raltitrexed (magenta square) at 50 °C resulting in an EC_50_ of 0.75±0.2 nM. Data are provided as two individual data points from one test occasion.

CETSA is based on measurements of remaining soluble target protein against a background of thermally denatured and precipitated proteins following a heat challenge^10^,^14^. To enable large-scale screening and automation we developed a no-wash immunoassay for TS using AlphaScreen technology in 384-well plates (see Supplementary Figs 1–6 and Supplementary Table 1). As outlined in Fig. 1a the assay workflow starts with a pre-incubation of K562 cells with library compounds or controls to allow cellular uptake, potential compound metabolism and binding to TS. The treated samples in the plates are next transiently heated in a PCR machine, resulting in denaturation and precipitation of intracellular TS unless stabilized by ligand. After cooling to room temperature the cells are lyzed and the remaining (stabilized) levels of TS are measured.

We validated the assay by investigating the response to two drugs of structurally different classes, that is, floxuridine and raltitrexed. Both drugs require intracellular enzymatic conversion prior to high-affinity TS binding^17^. Pyrimidine-based inhibitors, such as floxuridine, bind to TS as the corresponding monophosphate, whereas folate-based drugs, such as raltitrexed, are polyglutamylated and bind TS in a ternary complex with 2′-deoxyuridine 5′-monophosphate (dUMP). Two assay formats were employed for validation. First, the heating was done at a series of different temperatures at a fixed compound concentration to establish aggregation temperature (*T*_agg_) curves (Fig. 1b). As expected both drugs resulted in substantial shifts of the thermal stability of TS, thus confirming cellular uptake and intracellular enzymatic conversion to the active forms that bind TS. Based on these curves, 50 °C was selected for further characterization in isothermal dose-response fingerprint (ITDRF_CETSA_) experiments. In these experiments the compound concentration is titrated during the pre-incubation, after which all samples are heated to the same temperature (Fig. 1c,d). Both drugs showed dose-dependent stabilization of TS with half maximal effective concentration (EC_50_) values in the sub-nM range. Data from parallel experiments using quantitative western blots for assessment of stabilized TS confirmed a significant shift in *T*_agg_ in the presence of 5 μM of either of these drugs as well as potent dose-dependent stabilization (Supplementary Fig. 7). No change in total TS levels was observable at 5 μM concentration following a 2 h pre-incubation time in the K562 cells, demonstrating that the thermal stabilization data were not influenced by drug-induced changes in total protein levels under these conditions.

### Small molecule library screening and hit confirmation

**Figure 2:**
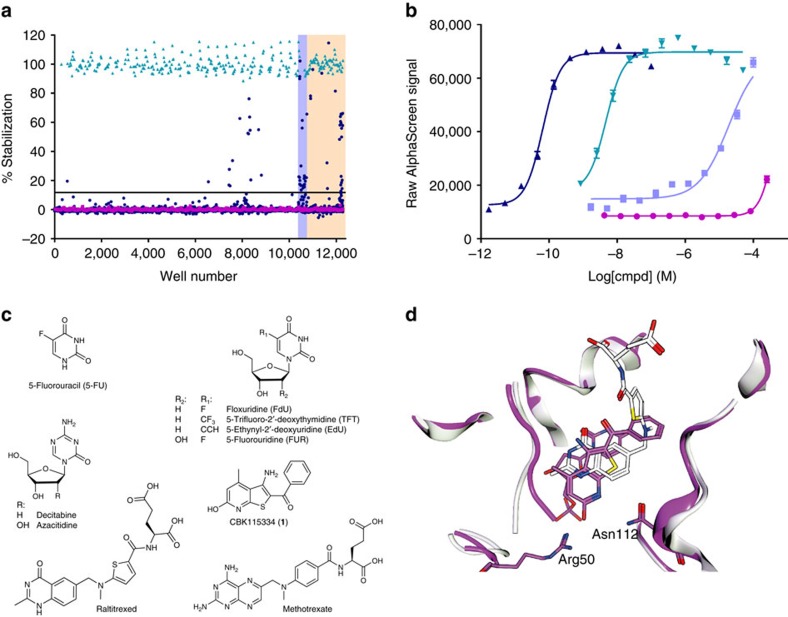
Primary screen using CETSA to measure target engagement of human thymidylate synthase. (**a**) Scatter plot illustrating normalized screen data, where 0% corresponds to the TS signal observed in the presence of DMSO only (magenta square) and 100% corresponds to the TS signal observed in the presence of 100 nM raltitrexed (green triangle). Data for library compounds at a concentration of 50 μM are shown in blue (blue circle). The hit limit was calculated based on the average plus three standard deviations for the library compounds and is illustrated as a black solid line at 11.7%. The locations of the Prestwick drug set (yellow) and a nucleoside subset (purple) are highlighted. (**b**) ITDRF_CETSA_ data illustrating the ranking of floxuridine (blue upwards triangle), 5-fluorouridine (FUR) (green downwards triangle), and 5-FU (lavender blue square) after 2 h of preincubation time. Data are also included for CBK115334 (magenta circle).The solid lines represent best fits to a saturation binding curve resulting in an apparent EC_50_ of TS at a concentration of 65±9 pM, 47±15 nM, 19±4 μM and 0.46±0.08 mM, respectively. Data are provided as the average and s.e.m. from one independent hit confirmation experiment done in quadruplicate. (**c**) Structures of known drugs and hit compounds discussed in the main text. (**d**) Structure of CBK115334 (magenta) and dUMP bound to TS, shown overlayed on the structure of the complex of raltitrexed (white) and dUMP (PDB 1HVY).

We screened a library of 10,928 compounds at Chemical Biology Consortium Sweden (CBCS; www.cbcs.se) using the TS assay described above. The library includes a structurally-diverse selection of lead-like compounds^20^, nucleosides and known drugs (see Methods section for details on the library). These latter subsets include folate and nucleoside-based drugs known to act on TS, suppress thymidine incorporation into DNA and reduce cell proliferation^16^,^21^. A schematic outline of the screen logistics is available in Supplementary Fig. 8. Screening was performed at a compound concentration of 50 μM and resulted in a reproducible response to the controls and the appearance of several stabilizing compounds (Fig. 2a). Additional graphs illustrating the screen performance and statistics are available in Supplementary Fig. 9 and Supplementary Table 2, respectively. The campaign involved one day of screening, with the AlphaScreen readings done the following morning to ensure equilibration of the antibody recognition.

The threshold for active solutions was calculated at 11.7% stabilization and resulted in 65 hits (Supplementary Table 3). Solutions for 63 of these were available for cherry-picking from our vial-based compound stores, that is, a different source intended for long-term storage. The activities of these solutions were examined in ITDRF_CETSA_ experiments to confirm the screen results (Supplementary Table 3). The majority of compounds with an apparent stabilization above 30% in the primary screen confirmed activity. We also found that 12 out of the 15 vial-based solutions that failed to reproduce activity (highlighted at the bottom of Supplementary Table 3) had been contaminated with highly active compounds because of insufficient tip washing during the transfer from vials to screen library plates (Supplementary Fig. 10). Consequently these hits reproducibly confirmed activity when tested from the contaminated source plates, while the original solutions were inactive. Taken together the confirmation rate was 90% for hits yielding more than 30% apparent thermal stabilization of TS in the original screen.

### Fluoropyrimidines, anti-folates and their analogs

The majority of confirmed hits were pyrimidine-based nucleosides and analogs thereof. At the top of the list were substituted 2′-deoxyuridines, including floxuridine (three independent occurrences in the library), 5-trifluoro-2′-deoxythymidine (TFT), and 5-ethynyl-2′-deoxyuridine (EdU) (Fig. 2b and Supplementary Table 3). All of these are known to be taken up and metabolized intracellularly to the active monophosphate forms that interact with TS^16^,^17^. Novel findings among the nucleosides included the two drugs azacitidine and its deoxyribose analog decitabine, as well as two purine nucleosides (8-bromoadenosine and 8-allyloxy-N2-isobutyryl-2′-deoxyguanosine).

With regards to folate analogs, methotrexate was present at two instances in the library, both as a racemate and as its L form. As expected both appeared as strong hits. The CETSA screen also identified two other marketed drugs, triamterene, a sodium channel inhibitor used to treat hypertension, and pyrimethamine, an inhibitor of dihydrofolate reductase from *Plasmodium falciparum* that is used to treat malaria. They have related structures and can potentially act as folic acid antagonists^22^, but they have not been previously shown to bind TS. Given the scarcity of anti-folates in the hit list we also looked whether there were any obvious false negatives in the screen and confirmed this was not the case (Supplementary Tables 4 and 5).

### CBK115334 as a novel TS inhibitor

Besides the pyrimidine- and folate-based inhibitors there were 17 additional weak hits of different chemical classes (Supplementary Table 3). We investigated one of these, CBK115334 or 3-amino-2-benzoyl-4-methylthieno(2,3-b)pyridin-6-ol (**1**), which was chemically distinct from known TS inhibitors (Fig. 2c and Supplementary Fig. 11). It also appeared for the first time as a hit in our screens (Supplementary Table 6). Confirmatory data were obtained using CETSA on K562 cell lysates, which demonstrated a 3.7 °C shift at 200 μM (Supplementary Fig. 12). When applied to isolated recombinant human TS, **1** showed a 2.6 °C shift at 25 μM and a 5.2 °C shift at 100 μM and confirmed binding in the low μM range using surface plasmon resonance (Supplementary Fig. 12). We tested whether binding affected enzymatic activity of TS *in vitro* and observed 60% inhibition at 10 μM concentration and near complete inhibition at 100 μM (Supplementary Fig. 12). A crystal structure of TS with **1** revealed that the compound binds the active site of TS occupying the folate-binding pocket (Fig. 2d). The binding involves *π*–*π* stacking interactions with the substrate dUMP and polar interactions with residues lining the catalytic cavity (Asn112 and Arg50 in particular). This constitutes a novel mode of binding as compared with other anti-folates occupying this space^23^,^24^. Finally, we investigated the impact on cell proliferation in K562 cells. A clear impact was seen with a half-maximal inhibitory concentration (IC_50_) value just below 100 mM (Supplementary Fig. 12), in line with the weak CETSA response.

### Addressing kinetics of compound transport and metabolism

**Figure 3:**
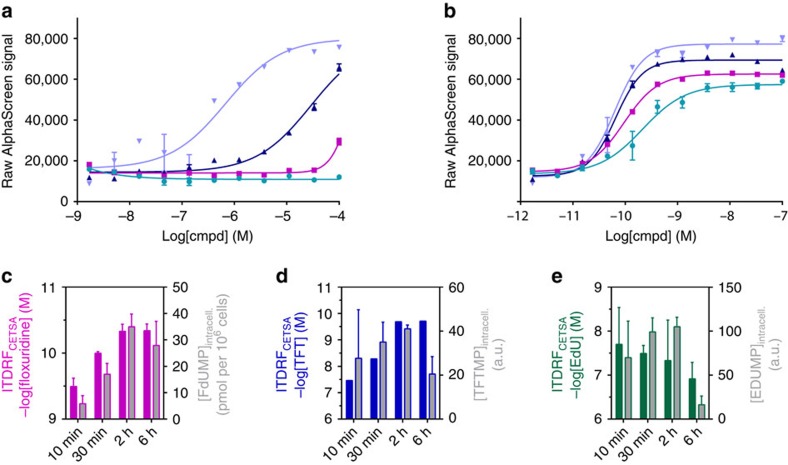
Time dependence of target engagement and correlation with the appearance of intracellular active metabolites. (**a**) Representative ITDRF_CETSA_ curves for 5-fluorouracil as a function of preincubation time in K562 cells; 10 min (green circle), 30 min (magenta square), 2 h (blue upwards triangle) and 6 h (lavender blue downwards triangle). The solid lines represent best fits to a saturation binding curve function to yield ITDRF_CETSA_ values for half-maximal stabilization of TS. Data are provided as the average and s.e.m. from experiments done in quadruplicate at a single test occasion. (**b**) The corresponding ITDRF_CETSA_ data for floxuridine. (**c**) Half-maximal stabilization of TS (magenta) and intracellular concentration of FdUMP (grey) as a function of preincubation time with floxuridine. The CETSA data are presented as the average and range from two independent experiments. The LC–MS/MS data are provided as the average and s.e.m. from experiments done at three different occasions. (**d**) The corresponding data for TFT (blue) and TFTMP (grey). (**e**) The corresponding data for EdU (green) and EdUMP (grey).

We performed time-traces of the ITDRF_CETSA_ experiments, that is, by varying the time during which cells were exposed to compound prior to the heating step. ITDRF_CETSA_ data obtained after various pre-incubation times are shown in Fig. 3a,b for 5-fluorouracil (5-FU) and floxuridine, demonstrating several orders of magnitude lower potency for 5-FU. The corresponding data on additional nucleosides are available in Supplementary Fig. 13. To examine whether the observed target engagement coincides with appearance of the active forms of these compounds, we monitored levels of compounds and their anticipated active metabolites using liquid chromatography coupled to tandem mass spectrometry (LC–MS/MS)^25^,^26^. Intracellular and extracellular concentrations as a function of incubation time are shown in Supplementary Fig. 14 for selected nucleosides and their corresponding monophosphate species.

The cellular import and metabolic activation of floxuridine and 5-FU to generate the common active species 5-fluoro-2′-deoxyuridine 5′-monophosphate (FdUMP) require different enzymatic pathways^16^,^17^,^27^. CETSA data for floxuridine showed stabilization at low nM concentrations after only 10 min of pre-incubation (Fig. 3b). The potency improved during the first 2 h and persisted throughout the experiment. This time trace was consistent with the intracellular appearance of FdUMP, which was measureable already after 10 min and increased during the first hours of incubation (Fig. 3c). However, for 5-FU the CETSA response increased slowly in the first 6 h (Fig. 3a), with undetectable intracellular levels of FdUMP (Supplementary Fig. 14). Meanwhile the concentration of 5-FU in cells and media remained relatively constant at all time points, thus demonstrating a fast cellular uptake (Supplementary Fig. 14). We hence conclude that the enzymatic conversion of 5-FU to FdUMP is much slower than for floxuridine in K562 cells under these conditions and that the enzymatic conversion to the active species is mirrored by the CETSA responses.

EdU and TFT are structurally related to floxuridine differing only at position 5 of the uracil moiety (Fig. 2c). Although their primary activity on cell viability is believed to result from their misincorporation into DNA, they are also known to inhibit TS following intracellular phosphorylation^28^,^29^. The uptake and metabolism of TFT, as well as its CETSA time trace, was similar to that observed for floxuridine (Fig. 3d). This was consistent with a build-up of 5-trifluoro-2′-deoxythymidine 5′-monophosphate (TFTMP) and binding to TS in the first hours, generating full target engagement after 2 h of incubation. TFTMP is known to be a tight-binding inhibitor that forms covalent complexes with TS also in absence of the folate-based cofactor^30^,^31^,^32^, in line with the observation of a persistent target engagement as measured by CETSA. EdU behaved differently with a more rapid uptake and faster decay of both the extracellular nucleoside and the active form 5-ethynyl-2′-deoxyuridine 5′-monophosphate (EdUMP) (Fig. 3e). The CETSA response was consistent with the fast uptake and activation with an early maximal response that then decayed slightly after the first 2 h, presumably due to the disappearance of the active species.

### Phosphorylation and deamination of decitabine

An unexpected hit in the screen was decitabine, which primarily acts as an inhibitor of DNA methyltransferase^33^. The identification of a 2′-deoxycytidine analog as a hit was surprising, but reinforced by the concurrent appearance of the corresponding ribose azacitidine (Fig. 2c). To shed further light on the generation of the active compound, TS stabilization by decitabine itself was first investigated in K562 cell lysates, where activating metabolism is lower because of significant dilution of intracellular enzymes and their substrates. As shown in Supplementary Fig. 15 TS was not stabilized by decitabine in treated lysates. Likewise decitabine did not stabilize recombinant TS in a thermal shift assay, in line with observations for other nucleosides including deoxyuridine, floxuridine and TFT (Supplementary Fig. 15).

**Figure 4:**
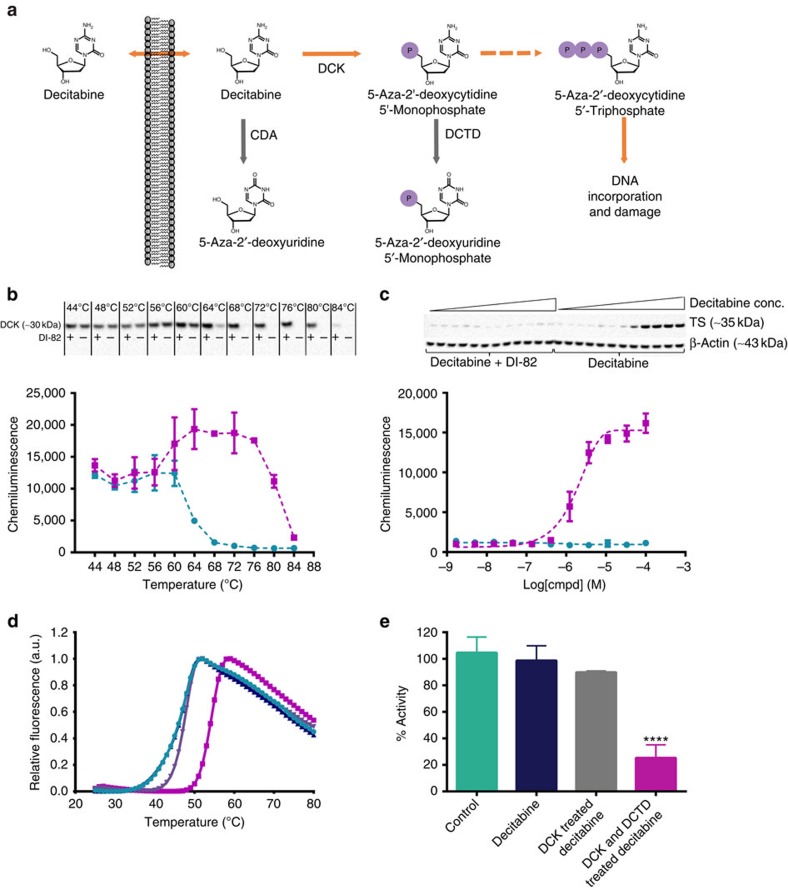
Target engagement by decitabine is dependent on its metabolic activation. (**a**) Schematic overview of decitabine treatment, cellular uptake and intracellular metabolic conversion. After uptake decitabine is phosphorylated to form 5-aza-2′-deoxycytidine 5′-monophosphate by DCK. This compound is further phosphorylated in two steps to yield the triphosphate that is incorporated into DNA. Cytidine deaminase (CDA) and DCTD are known to be involved in the metabolism and clearance of decitabine^39^. (**b**) *T*_agg_ experiments for CDK in the absence (green circle) and presence of 200 μM of the DCK inhibitor DI-82 (magenta square). Above the graphs are the chemiluminescence data (full blots are available in Supplementary Fig. 16). The experiments were performed in K562 cells at two independent occasions. (**c**) ITDRF_CETSA_ data for decitabine in the absence (magenta square) and presence of 200 μM of the DCK inhibitor DI-82 (green circle). Full blots are available in Supplementary Fig. 16. The experiments were performed in K562 cells at two independent occasions. (**d**) Normalized thermal shift assay response for recombinant human TS in the absence (magenta square) and presence of 1 mM decitabine without prior enzyme treatment (blue upwards triangle), following DCK treatment (lavender blue downwards triangle) and following treatment with both DCK and DCTD (magenta square). The data are shown as the average and s.e.m. from triplicate samples at one test occasion. (**e**) Enzyme inhibition data for TS in the presence of control and enzymatically treated decitabine samples. The data are shown as the average and s.e.m. from triplicate samples at one test occasion.

The structural analogy to the known nucleoside-based inhibitors of TS triggered the question as to whether decitabine is also phosphorylated, and potentially also deaminated, to generate a TS ligand (Fig. 4a). To investigate the importance of phosphorylation we performed ITDRF_CETSA_ experiments in the presence of DI-82 (ref. 34). This compound is a potent inhibitor of deoxycytidine kinase (DCK) (Fig. 4b), which is required for formation of decitabine monophosphate^35^,^36^. Dose-dependent stabilization of TS was confirmed in the absence of DI-82, whereas its presence at 200 μM completely blunted the ability of decitabine to bind TS (Fig. 4c). However, studies using recombinant DCK to generate decitabine monophosphate resulted in only a marginal stabilization of TS (Fig. 4d). The sample was therefore additionally treated with deoxycytidylate deaminase (DCTD), which is known to deaminate decitabine monophosphate^37^, resulting in a thermal shift of nearly 10 °C (Fig. 4d). The inhibitory capacity of these samples mirrored these data, that is, minor inhibition was observed after DCK treatment in the TS enzymatic assay, whereas near full inhibition appeared after treatment with both DCK and DCTD (Fig. 4e). Taken together with the structural analogy to the natural substrate of TS these data strongly infer that the TS ligand is 5-aza-2′-deoxyuridine 5′-monophosphate, that is, the expected product of phosphorylation and deamination of decitabine.

## Discussion

Target engagement is essential for efficacy of targeted therapies and validation of new chemical probes^2^,^4^,^15^. These validating experiments are ideally performed for many representatives within a chemical series to allow comparisons of structure–activity relationships. To push towards the goal of having a procedure amenable to automation and screening we applied CETSA for assessment of intracellular target engagement at the stage of primary screening. Prior to this work the methodology had been applied on a growing number of drugs and chemical probes^10^,^11^,^12^,^13^,^14^, but it remained challenging to apply to large chemical libraries.

To achieve this evaluation, we developed a homogeneous CETSA and applied it to screening in live, non-engineered cells expressing thymidylate synthase, which is targeted by several different chemical classes of drugs in clinical use^16^,^17^. Importantly, the screen identified all drugs within the test library that act on TS, as well as novel compounds capable of binding and inhibiting this enzyme. Collectively the known drugs and new inhibitors span over a broad range of affinities. Amongst the new hits was **1**, a μM inhibitor of the purified enzyme with sufficient cell penetration to result in intracellular target engagement and anti-proliferative effects in the high μM range. The binding mode of **1** to TS is partly new and it thus provides a potential starting point for further chemistry optimization.

Several other marketed drugs not previously known to inhibit TS emerged as hits, including triamterene, pyrimethamine and decitabine. As these are clinically-used compounds it is of interest to understand whether there are instances where the interaction with TS plays a role in either efficacy or toxicity. The identification of decitabine illustrates the relevance of monitoring target binding in live cells as this finding was dependent on active cellular metabolism. Decitabine itself was largely inactive on the protein such that, in analogy to the already known uridine-based inhibitors, enzymatic conversion to an active species is a prerequisite for observation of binding. We showed that this conversion does not take place to a significant extent in cell lysates, but can be reproduced with the *in vitro* application of enzymes for phosphorylation and deamination to yield the substrate analog 5-aza-2′-deoxyuridine 5′-monophosphate. Although our data give strong support that a metabolite of decitabine yield significant cellular inhibition of TS, further studies are required to determine whether this is relevant for polypharmacology or toxicity at typical therapeutic doses.

Time-traces of ITDRF_CETSA_ were used to analyze different scenarios for cellular target engagement, thereby integrating aspects of drug transport and metabolism. Combination of these results with measurements of intracellular drug and metabolite concentrations allowed for a comprehensive dissection of cellular drug kinetics. Overall, the intracellular concentrations of the active species of the drugs correlated with the observed target engagement, consistent with the notion that CETSA directly reports on target binding. The combination of high-throughput target engagement studies with LC–MS/MS measurements of intracellular concentrations of drugs and metabolites constitutes a new paradigm for hit validation and optimization in the discovery of chemical probes and drugs. Importantly, CETSA is applicable to studies in native cells and tissue samples^10^,^14^. Thus the basic scenarios for compound metabolism and target engagement derived from these cell culture studies should be possible to translate towards studies of activities and resistance development to drugs in man. Of particular interest in this regard was the observation that we nearly missed the identification of 5-FU in the screen because of the relatively slow appearance of target engagement. It will be interesting to extend these experiments to patient cells.

The present work demonstrates that CETSA constitutes a robust high-throughput screening strategy that allows for target proteins to be approached in their natural cellular environment. This is in contrast to the majority of targeted cellular HTS assays as these rely on overexpressed and tagged proteins. Since CETSA does not require engineered cells or compounds, it could be particularly attractive for screening in primary cells, tissues or patient-derived material. Our approach can be applied to a large number of different proteins, with the generic assay development path being established in this work. The work also introduces the combination of time-dependent ITDRF_CETSA_ and measurement of intracellular concentrations of metabolites as a stringent approach for hit validation, where the same assay format can be utilized and provide value throughout the drug discovery process.

## Methods

### Cell culture conditions in AlphaScreen-based experiments

Human myelogenous leukemia cell line K562 (ATCC no. CCL-243) were cultured in RPMI-1640 (SH30027.01, HyClone) supplemented with 10% fetal bovine serum (SV30160.03, HyClone), 0.3 g l^−1^ L-glutamine (G7513, Sigma-Aldrich) and 100 units ml^−1^ Penicillin-Streptomycin (P4333, Sigma-Aldrich). The same cell medium composition was used for all experiments unless otherwise stated.

### Development of an AlphaScreen-based assay for TS

Measurements of remaining levels of soluble TS in cell lysates were achieved based on an AlphaScreen-based assay. Establishment of this assay required the identification of a pair of antibodies that simultaneously recognize TS (Supplementary Fig. 1). Combinations of four mouse-derived and three rabbit-derived antibodies directed towards different epitopes of TS (see Supplementary Table 1) were tested for this ability. Four different conditions were tested for each pair, with two of those being the absence and presence of target protein. Given our previous experience of ligand induced quenching of protein target recognition by the antibody pair^14^ we also included a control containing an excess of dUMP with and without the additional presence of raltitrexed, which are known binders to the active sites of TS. Recombinant TS, diluted in 1 × AlphaLISA buffer (AL000F, PerkinElmer), was preincubated at room temperature in the presence of buffer only, 100 μM dUMP or 100 μM dUMP and 10 μM raltitrexed in a total volume of 4 μl in a ProxiPlate (#6008280, PerkinElmer). After this preincubation all 12 possible combinations of antibody pairs were added to the sample in a volume of 4 μl followed by incubation for 30 min at room temperature. A mix of AlphaScreen acceptor and donor beads was finally added in a volume of 4 μl under subdued light and allowed to incubate at room temperature for 2 h before reading in an Envision plate reader (PerkinElmer). Final concentrations of the reagents in the detection step were 2 nM recombinant TS, 2 nM of each antibody, 40 μg ml^−1^ AlphaScreen anti-mouse donor beads (#AS104D, PerkinElmer) and 10 μg ml^−1^ AlphaScreen anti-rabbit acceptor beads (#AL104C, PerkinElmer). The plates were sealed with TopSeal-A PLUS (6050185, PerkinElmer). The data were analyzed using microsoft excel and GraphPad Prism 6.

Four different antibody pair combinations based on sc-376161, WH0007298M1, 15047-1-AP and D5B3 (Supplementary Table 1) were selected from the antibody screen to study the kinetics of their recognition of TS in cell lysate. Two batches of 7.5 million K562 cells per ml in supplemented cell culture medium were prepared to serve as max and min controls. One culture was left at room temperature and the other was heated to 52 °C for 3 min in a PCR machine (TECHNE TC-PLUS thermal cycler). Both batches of cells were then lysed by the addition of an equal volume of 2X AlphaScreen SureFire Lysis Buffer (TGRLB100ML, PerkinElmer). After thorough mixing, 4 μl aliquots of the lysates were transferred to a ProxiPlate and detected and analyzed as described above except that the bead incubation was performed at 2 h, 6 h and overnight.

The sc-376161 and 15047-1-AP antibodies were titrated to match their concentrations with the AlphaScreen bead concentrations, that is, to ensure they do not exceed concentrations where hook effects are observed. Fifteen million K562 cells per ml were prepared in supplemented cell culture medium and lysed as described above. Aliquots of 4 μl were transferred to a ProxiPlate followed by the addition of 4 μl of a mix of different concentrations of the two antibodies (final concentrations of each antibody in the detection varied between 0 and 10 nM). Detection and analysis was done as described above, except the bead incubation was performed overnight.

Optimization of cell numbers was achieved by serial dilution of a cell suspension of K562 cells in supplemented cell culture medium. Each sample was then split into two aliquots, which were either kept at room temperature or heated to 52 °C as described above. Both aliquots were then lysed as described above. After thorough mixing, 3 μl of the lysates were transferred to a ProxiPlate followed by the addition of 6 μl of a mix of antibodies and AlphaScreen acceptor and donor beads in AlphaLISA buffer under subdued light. Detection and analysis was achieved as described above except the antibody concentrations were modified to 1 nM 15047-1-AP and 0.4 nM sc-376161.

The control experiment, in which recombinant TS was seeded to cell lysates was prepared based on a serial dilution of recombinant human TS. Dilutions were done in equal volumes of supplemented cell medium and 2 × AlphaScreen SureFire Lysis Buffer. K562 cells at a cell density of 2 million cells per ml were lysed as described above and split in two samples, which were either kept at room temperature or heated to 52 °C as described above. A 10 μl aliquot of each TS dilution was then added to the same volume of each of the two lysates as well as to the mixture of cell medium and lysis buffer. A 3 μl aliquot of each sample was then transferred to a ProxiPlate and detected and analyzed as described above.

### Thermal aggregation experiments using AlphaScreen

Floxuridine and raltitrexed were diluted from dimethyl sulfoxide (DMSO) stock solutions to concentrations of 30 μM and 20 μM respectively in supplemented cell culture medium (final DMSO content 1%). These solutions were transferred in a volume of 10 μl to a skirted Twin.tec PCR 96-wellplate (0030 128 672, Eppendorf). A suspension of K562 cells in a volume of 10 μl and a density of 10 million cells per ml were then added to all wells. The PCR plates containing the compounds and cells were sealed with a breathable plate seal (3345, Corning) and incubated for 2 h in a humidified incubator at 37 °C and 5% CO_2_. The cells were then transiently heated to different temperatures ranging from 40 °C to 86 °C for 3 min, followed by a controlled cooling to 20 °C for 1 min using a real-time PCR machine (ProFlex, Applied Biosystems). After the heating step the plate was centrifuged briefly (1,000 × *g* for 1 min) followed by lysis of the heated cells by the addition of 20 μl of 2 × AlphaScreen SureFire Lysis Buffer using a Flexdrop IV (PerkinElmer). To ensure sufficient lysis the cell lysates were mixed by 10 repetitive aspiration and dispensing cycles using a Bravo liquid handling platform (Agilent). The lysates (3 μl) were then transferred to 384-well ProxiPlates followed by the addition of 6 μl of a mix of antibodies and AlphaScreen acceptor and donor beads in AlphaLISA buffer under subdued light. Final concentrations of the assay reagents in the detection step were 1 nM rabbit polyclonal anti-TS IgG (15047-1-AP, Proteintech), 0.4 nM mouse monoclonal anti-TS IgG (sc-376161, Santa Cruz), 40 μg ml^−1^ AlphaScreen anti-mouse donor beads and 10 μg ml^−1^ AlphaScreen anti-rabbit acceptor beads. The plates were sealed with TopSeal-A PLUS and incubated over night at room temperature prior to detection in an Envision plate reader. The data were analyzed using microsoft excel and GraphPad Prism 6.

### Composition and storage of the primary screening set

The library of compounds applied in this screening campaign consists of 10,928 compounds and is part of the primary screening set at CBCS. The majority of these compounds was donated by Biovitrum AB and originates from both in-house and commercial sources. Compounds included in the primary screening set were selected to represent a diverse selection of a larger set of 65,000 compounds, while keeping a certain depth to allow crude structure–activity relationship studies. The selection was also biased towards lead-like and drug-like profiles with regards to molecular weight, hydrogen bond donors/acceptors and LogP^20^. The library also includes a nucleoside set from Berry & Associates and a set of approved drugs from Prestwick. Compound stock solutions at 10 mM in DMSO are stored frozen at approximately −20 °C in individual capped tubes in REMP 96 Storage Tube Racks. The racks are stored in a REMP Small-Size Store, which allows cherrypicking while the solutions are still frozen to minimize repetitive freeze-thaw cycles. For screening purposes the compound solutions have been replicated from the REMP racks to Labcyte 384 LDV plates (LP-0200) and then further into Labcyte 1536 HighBase plates (LP-03730) to enable dispensing using acoustic liquid handling equipment.

### Compound handling

Assay ready plates were prepared by transferring 200 nl of the 10 mM DMSO solutions of compounds and controls by means of acoustic dispensing (Echo 550, Labcyte) to 384-well polypropylene plates (784201, Greiner). Compounds were placed in columns 1–22. DMSO controls were placed in column 23 and raltitrexed controls were placed in column 24. The assay ready plates were heat sealed with a Peelable Aluminium seal (24210-001 Agilent) using a thermal microplate sealer (PlateLoc, Agilent) and stored at −20 °C until use. At the day of the experiment the plates were allowed to thaw for 30 min followed by a brief centrifugation step (1,000 × *g* for 1 min) prior to removal of the seal. The compounds were then diluted with 20 μl supplemented cell culture medium using a Multidrop Combi reagent dispenser (Thermo Scientific). Finally 5 μl of the diluted compounds were transferred to a 384 well hardshell PCR plate (HSR480, BIORAD) using a Bravo liquid handling platform equipped with a 384-well head (Agilent). The final concentrations in the incubation with cells (see below) were 50 μM of test compounds and 100 nM of the positive control raltitrexed. The final concentration of DMSO in the assay was 0.5% in all samples.

For the ITDRF_CETSA_ experiments 11-point dose-response curves with three-fold difference in concentration between wells were generated using the Bravo liquid handling system (all serial dilutions were done in 100% DMSO). The final highest concentrations of the test compounds in the incubation with cells were ranging from 100 nM–50 μM depending on estimated potency. The concentration of DMSO was 0.5% in all samples. The final concentration of the positive control raltitrexed was 100 nM. The assay ready plates were prepared as outlined above for the screen.

### Screening and dose-response characterization by AlphaScreen

The screen procedure started with the addition of 5 μl of a suspension of K562 cells at a density of 10 million cells per ml to all wells of a 384 well hardshell PCR plate (HSR480, BIORAD) using an electronic multichannel pipette (Biohit). The plates were then heat sealed with Peelable Aluminium seal in a thermal microplate sealer (PlateLoc, Agilent) and allowed to incubate for 2 h in an incubator at 37 °C and 5% CO_2_. For the time-course experiments the incubation times were altered to include also 10 min, 30 min and 6 h. To allow gas exchange during the longer incubation times these plates were instead sealed with at breathable plate seal (3345, Corning). After the incubation step the plates were transiently heated at 50 °C for 3 min followed by a controlled cooling to 20 °C for 1 min using a real-time PCR machine (LightCycler480 system, Roche). Plate handling was then as described above for the thermal aggregation experiments. The data were analyzed using microsoft excel and GraphPad Prism 6.

### Screen and ITDRF_CETSA_ data analysis

Screen data were imported into microsoft excel and normalized for each compound based on the negative and positive controls on each plate, that is, with the response in the presence of DMSO defining 0% stabilization and the response in the presence of 100 nM raltitrexed defining 100% stabilization. A calculation of the average and standard error of means for each set of controls also allowed an illustration of how these responses varied over the 32 screening plates. The Z′ factor^38^ is commonly used as a measure of how well the assay separates between the controls and this was calculated as described based on the calculated averages and standard deviations of the controls on a per plate basis. For the *T*_agg_ shift and the ITDRF_CETSA_ experiments the data were analyzed in GraphPad Prism using the Boltzmann sigmoid equation and the saturation binding curve (rectangular hyperbola; binding isotherm) function, respectively. As already discussed^14^ these methods make use of equilibrium models for data analysis although the methodology depends on the irreversible aggregation of denatured material. For this reason we refer to the observed responses as apparent and isothermal dose-response fingerprints and are careful with any quantitative interpretations, being well aware of their dependency on experimental conditions. Experiments are on-going to address the quantitative interpretation of CETSA data.

### Measurements of identity and purity of test compound solutions

Assessments of identity and purity of the test solutions that were used for hit confirmation purposes, that is, those being stored in REMP vials, was done by means of high-pressure liquid chromatography coupled to mass spectrometry (HPLC–MS). A small aliquot of each test solution (2 μl of a 10 mM solution) was placed in a 96-well plate (267245, Nunc) and diluted with 20 μl of methanol. The plate was then placed in an Agilent 1,100 HPLC UV/MS with electrospray ionization (ESI+). The HPLC method was based on an ACE C8 3 μm column (3.0 × 50 mm) and a mobile phase (CH_3_CN)/(0.1% TFA/H_2_O). All solvents were HPLC grade and absorbance was monitored at 220 nm. Compounds that did not give satisfactory data were re-analyzed using a method based on a Waters XBridge C18 3.5 μm column (3.0 × 50 mm), 3.5 min gradient mobile phase (CH_3_CN)/(10 mM NH_4_HCO_3_/H_2_O). The instrument software was used to integrate the UV response for each peak and provided a list of the peaks and their associated masses. The estimated purity was calculated based on the integrated area for the expected mass compared with the areas of all other peaks. The result was manually controlled and if there were deviations from the expected outcome a meticulous investigation of the UV-response and MS was performed.

### Cell viability assay

A concentration–response curve of CBK115334 was generated using the Bravo liquid handling system (serial dilution of a 50 mM stock solution was done in 100% DMSO). A total of 150 nl of the serially diluted solutions and controls (positive control 0.67 mM staurosporine and negative control DMSO) were transferred to a white 384-well assay plate (3570, Corning) by means of acoustic dispensing (Echo 550, Labcyte). A Multidrop reagent dispenser (Thermo Scientific) was used to dispense 30 μl of a K562 cell suspension at a density of 33 × 10^3^ cells per ml in supplemented cell culture medium. The cells were incubated at 37 °C in the presence of 5% CO_2_ for 72 h before addition of 30 μl CellTiter-Glo Luminescent Cell Viability Assay reagent (Promega) using a Multidrop Combi reagent dispenser (Thermo Scientific). The plate was placed on a plate shaker for 15 min prior to detection of the luminescence signal in an Envision plate reader (PerkinElmer). The final highest concentration of CBK115334 in the incubation with cells was 250 μM and the final concentration of the positive control staurosporine was 3 μM. All samples contained 0.5% DMSO. The data were analyzed using microsoft excel and GraphPad Prism 6.

### Chemicals and buffers in western blot-based experiments

The cell lysis buffer contained 100 mM 4-(2-hydroxyethyl)-1-piperazineethanesulfonic acid (HEPES, pH 7.5), 1 mM Tris-(2-carboxyethyl)phosphine hydrochloride (TCEP) and 10 mM magnesium chloride (Sigma-Aldrich) supplemented with complete (EDTA-free) protease inhibitor cocktail from Roche (Switzerland). Tris-buffered saline with tween (TBST) buffer (150 mM NaCl, 0.05% (v/v) Tween-20, 50 mM Tris-HCl buffer at pH 7.6) was prepared by dissolving TBS-TWEEN tablets obtained from Merck KGaA (Darmstadt, Germany) in ddH_2_O. The blocking buffer consisted of 5% (w/v) non-fat milk (Semper AB, Sundbyberg, Sweden) diluted in tris-buffered saline with tween. Hank's Balanced Salt Solution (HBSS) was from Gibco/Life Technologies. Raltitrexed monohydrate, dUMP and decitabine was purchased from Sigma-Aldrich and Selleckchem, respectively. DI-82 was kindly provided by Prof. Caius G. Radu and Raymond M. Gipson at the Department of Molecular and Medical Pharmacology, University of California, Los Angeles.

### Cell lines and cultures in western blot-based experiments

Human cell line K562 (ATCC no. CCL-243) was cultured in RPMI-1640 medium (Sigma-Aldrich) supplemented with 0.3 g l^−1^ L-glutamine and 10% fetal bovine serum (FBS, Gibco/Life Technologies, Carlsbad, CA, USA), 100 units per ml penicillin and 100 units per ml streptomycin (Gibco/Life Technologies). Short-term passages (<20) were used for experiments.

### Cell lysate thermal shift experiments

For the cell lysate thermal shift experiments, cultured K562 cells were harvested and washed with Hank's Balanced Salt Solution. The cells were diluted in lysis buffer supplemented with complete protease inhibitor cocktail. The cell suspensions were freeze-thawed three times using liquid nitrogen and passed through a 27″ gauge needle five times. The soluble fraction (lysate) was separated from the cell debris by centrifugation at 20,000 × *g* for 20 min at 4 °C. For the thermal aggregation curve experiments cell lysates were diluted with lysis buffer supplemented with 200 μM dUMP and divided into two aliquots, with one aliquot being treated with ligand and the other aliquot with vehicle (control). After 10 min incubation at room temperature the respective lysates were divided into smaller (50 μl) aliquots and heated individually at different temperatures for 3 min in a Veriti thermal cycler (Applied Biosystems/Life Technologies) followed by cooling for 3 min at room temperature. The heated lysates were centrifuged at 20,000 × *g* for 20 min at 4 °C in order to separate the soluble fractions from precipitates. The supernatants containing the remaining soluble proteins were transferred to new 0.2 ml microtubes and analyzed by sodium dodecyl sulfate polyacrylamide gel electrophoresis (SDS-PAGE) followed by western blot analysis.

For the in-cell experiments K562 cells were harvested and resuspended with culture medium to a cell density of 5 million cells per ml and seeded into T25 flasks. Cells were treated with either raltitrexed, floxuridine, DI-82 or vehicle (DMSO) for 2 h in an incubator at 37 °C and 5% CO_2_. The cell suspensions were then divided into 100 μl aliquots in 0.2 ml tubes and heated at designated temperatures ranging from 40 to 84 °C for 3 min in a Veriti thermal cycler (Life Technologies) followed by 3 min of cooling at room temperature. The heat-treated cell suspensions were freeze-thawed three times using liquid nitrogen and a heating block set at 25 °C. Tubes were gently vortexed between the freeze-thaw cycles. The resulting cell lysates were centrifuged at 20,000 × *g* for 20 min at 4 °C. The supernatant was removed from the cell debris and aggregates and the remaining soluble TS was analyzed using western blot.

For the ITDRF_CETSA_ in cell experiments, raltitrexed and floxuridine were serially diluted to generate an 11 point dose–response curve with three-fold difference in concentration between each point. K562 cells were treated with each respective compound concentrations and one vehicle as control in 100 μl aliquots in 0.2 ml tubes for 2 h in an incubator at 37 °C and 5% CO_2_. The cell aliquots were heated at 50 °C and analyzed with western blot following the procedure described above.

For the decitabine metabolism, decitabine was serially diluted to generate an 11 point dose–response curve and an ITDRF_CETSA_ experiment was performed as described above in absence and presence of 200 μM of the DCK inhibitor DI-82 (referred to as 12R in the main text of the original publication and DI-82 in the Supplementary Material)^34^.

### SDS-PAGE and western blot

NuPage Novex Bis-Tris 4–12% polyacrylamide gels with NuPAGE MES SDS running buffer (Life Technologies) were used for separation of proteins in the samples. Proteins were transferred to nitrocellulose membranes using the iBlot2 blotting system (Life Technologies). Primary antibodies anti-TS (D5B3) XP (Cell Signaling), anti-dCK (sc-393099), anti-β-actin (sc-69879); secondary goat anti-mouse HRP-IgG (sc-2055) and bovine anti-rabbit HRP-IgG (sc-2374) antibodies (Santa Cruz Biotechnology, Santa Cruz, CA, USA) were used for immunoblotting. All membranes were blocked with blocking buffer; standard transfer and western blot protocols recommended by the manufacturers (listed above) were used. All antibodies were diluted in blocking buffer. The membranes were developed using Clarity Western ECL substrate HRP-Substrate (Bio-Rad) according to the manufacturer's recommendations. Chemiluminescence intensities were detected and quantified using a ChemiDoc XRS+ imaging system (Bio-Rad) with Image Lab software (Bio-Rad).

### Expression and purification of human thymidylate synthase

The gene encoding human TS (NM_001071.2) was subcloned into the pNIC28-Bsa4 vector and expressed in Rosetta BL21-DE3 Escherichia coli (Novagen) in Terrific Broth media supplemented with 50 μg ml^−1^ of kanamycin and 34 μg ml^−1^ chloramphenicol. Cells were grown at 37 °C until OD_600 nm_ reached about 2.0 and induced with 0.5 mM isopropyl-beta-D-1-thiogalactopyranoside (IPTG) at 18 °C overnight. The cells were harvested by centrifugation at 4,500 × *g* for 15 min at 15 °C. The cell pellet was re-suspended in lysis buffer (100 mM HEPES, 500 mM NaCl, 10 mM imidazole, 10% (v/v) glycerol and 1 mM TCEP at pH 8.0) supplemented with 1:1,000 (v/v) EDTA-free protease inhibitor cocktail (Calbiochem) and 125 U ml^−1^ of Benzonase (Merck). Cells were lysed by sonication on ice at 70% amplitude, 3 s on/off for 3 min. The lysate was clarified by centrifugation at 47,000 × *g* for 25 min at 4 °C, and the supernatant was filtered through a 1.3 μm syringe filter to remove cell debris. The cell-free extract was loaded on a pre-equilibrated HisTrapTM HP column (GE Healthcare) in IMAC wash buffer 1 (20 mM HEPES, 500 mM NaCl, 10 mM imidazole, 10% (v/v) glycerol and 1 mM TCEP at pH 7.5) and subsequently washed with 20 column volumes (CVs) of IMAC wash buffer 1 and 15 CVs of IMAC wash buffer 2 (20 mM HEPES, 500 mM NaCl, 25 mM imidazole, 10% (v/v) glycerol and 1 mM TCEP at pH 7.5). Bound protein was eluted with 5 CVs of elution buffer (20 mM HEPES, 500 mM NaCl, 500 mM imidazole, 10% (v/v) glycerol and 1 mM TCEP at pH 7.5) and loaded onto a HiLoad 16/60 Superdex-200 column (GE Healthcare) pre-equilibrated with buffer (20 mM HEPES, 300 mM NaCl, 10% (v/v) glycerol, and 1 mM TCEP at pH 7.5). Based on Nu-PAGE gel results pure protein fractions were pooled and concentrated using a 50 kDa cutoff centrifugal driven filter concentrator (Sartorius Stedium Biotech). The protein concentration was determined by the absorbance at 280 nm using a Nanodrop spectrophotometer (Thermo Scientific).

### Thermal shift *in vitro* assay on recombinant protein

The assay was performed on the iCycler iQ Real Time PCR Detection System (Bio-Rad), using the 96-well thin-wall PCR plate (Bio-Rad). The experiment was conducted in a buffer containing 20 mM HEPES at pH 7.5 and 150 mM NaCl. A total volume of 25 μl solution containing 0.2 mg ml^−1^ protein, compounds and × 5 Sypro Orange dye (Invitrogen) diluted from 5,000 × stock was dispensed into the 96-well plate. The same amount of DMSO was added in the control wells. The plates were sealed with Microseal B adhesive sealer (Bio-Rad) and heated in iCycler from 25 to 80 °C (56 heating cycles in 28 min). Fluorescent filter used for Sypro Orange measurements was *λ*_excitation_=492 nm and *λ*_emission_=610 nm. The calculation of the midpoint of the curves (*T*_*m*_) was performed using the software package XLfit from IDBS within microsoft excel.

### TS enzyme inhibition assay

Enzymatic activity of recombinant human TS was measured spectrophotometrically at 340 nm by monitoring the absorbance change during the conversion of 5,10-methylenetetrahydrofolate to dihydrofolate using an Infinite M200 spectrometer (Tecan). Measurements were carried out at room temperature and in a buffer of 50 mM Tris at pH 7.5 and 150 mM NaCl. Initial velocities were measured with 250 nM of purified protein, 100 μM 5,10-methylenetetrahydrofolate and 100 μM dUMP in the presence of compounds using the same amount of DMSO as control. Initial rates and activity were analyzed with the software package Prism (GraphPad Software).

### Surface plasmon resonance

Recombinant human TS protein (25 μg ml^−1^ in a buffer of 10 mM sodium acetate at pH 5.0, 1 mM dUMP and 200 μM methotrexate) were captured on Sensor Chip S-CM5 via amine coupling to a level of ∼5,000 resonance units using Biacore T-200. Raltitrexed was used as a positive control to ensure that the protein remained active after immobilization and during the run. A concentration series (20 nM to 10 μM) of CBK115334 was injected over the prepared surface for 60 s and allowed to dissociate for 60 s with a flow rate of 70 μl min^−1^ at 25 °C. The assay buffer was 20 mM HEPES, 150 mM NaCl, pH 7.5 and 0.005% Tween-20 supplemented with 1% DMSO and 200 μM dUMP. Response data was processed using the BIAevaluation software. Responses were double referenced and solvent-corrected. The data sets were fitted to 1:1 steady-state model for determination of binding constants.

### Decitabine treatment with DCK and DCTD

Decitabine at a concentration of 1 mM was incubated with 1 mg ml^−1^ of recombinant DCK and 2.5 mM ATP in a buffer of 50 mM Tris at pH 7.5, 150 mM NaCl and 0.5 mM MgCl_2_ at room temperature for 60 min to generate the corresponding monophosphates. To probe for nucleotide deamination the samples were further treated with 1 mg ml^−1^ of recombinant DCTD in the presence of 1 mM ATP and 10 μM ZnCl_2_ for 20 min at room temperature. After incubation, the samples were heated at 95 °C for 10 min and centrifuged for 10 min at the highest speed at 4 °C using a benchtop centrifuge. The supernatant was tested without dilution in thermal shift *in vitro* assays and the TS enzyme inhibition assay. The supernatants from samples without nucleoside were used as the treatment controls.

### Crystallization and structure determination

TS protein crystallized in sitting drops comprising equal volume of protein (about 24 mg ml^−1^) and reservoir solution at 20 °C. The crystallization condition was composed of 0.1 M sodium cacodylate at pH 6.5 and 15% PEG 4,000. Crystals were soaked with 1 mM compound and 2 mM dUMP in cryo-protectant buffer containing 0.1 M sodium cacodylate at pH 6.5 and 25% PEG 4,000 and 10% DMSO for 15 min, followed by flash frozen in liquid nitrogen. Data collection was performed on beamline MX1 at Australian Synchrotron. X-ray diffraction data was collected at 100 K with a wavelength of 0.9537 Å. It should be noted that CBK115334 is subject to keto–enol isomerization. While the resolution is not sufficient to distinguish between these, the included illustrations are based on the enol form (both forms make interactions with Asn112 and Arg50, but with different donor–acceptor pairs). The structure was solved by molecular replacement using Phaser with the hTS-dUMP-raltitrexed structure (PDB code 1HVY) as the search model. Structure was refined with phenix refine. Ligand structures and restraints files were generated using eLBOW. In the TS-dUMP-CBK115334 complex structure, 90.43% of residues were in favoured regions and 7.92% of residues were in allowed regions. The data collection parameters and refinement statistics are summarized in Table 1. An image of the electron density map of the active site in the co-crystal structure is available in Supplementary Fig. 17.

### Intracellular compound and metabolite concentrations

Intracellular concentrations of compounds were measured as previously described^25^,^26^. Briefly, K562 cells were incubated with the compounds for a predefined time at 37 °C in a 5% CO_2_ atmosphere. After incubation, the cells were centrifuged (300 × *g* for 5 min) and a medium sample (supernatant) was collected and diluted 1:10 in a 50 nM warfarin solution (internal standard). The cells were washed with phosphate buffered salt solution and lysed with methanol. Methanol was evaporated and the cell samples were reconstituted in 50 nM warfarin. Samples were analyzed with LC–MS/MS with electrospray ionization in negative mode with transitions monitored as listed in Table 2.

## Additional information

**Accession codes:** The crystal structure of CBK115334 and dUMP in complex with TS has been deposited with the RCSB Protein Data Bank under the 5HS3 .

**How to cite this article:** Almqvist, H. *et al*. CETSA screening identifies known and novel thymidylate synthase inhibitors and slow intracellular activation of 5-fluorouracil. *Nat. Commun.* 7:11040 doi: 10.1038/ncomms11040 (2016).

## Supplementary Material

Supplementary Information Supplementary Figures 1-17, Supplementary Tables 1-6, Supplementary Note 1 and Supplementary References

## Figures and Tables

**Table 1:** X-ray diffraction data and refinement statistics.

	**TS-dUMP-CBK115334**
*Data collection*	
* *Space group	P4_3_2_1_2
* *Cell dimensions	
* a*, *b*, *c* (Å)	108.2, 108.2, 313.9
* α*, *β*, *γ* (°)	90, 90, 90
* *Resolution (Å)	30-3.1 (3.2–3.1)*
* R*_merge_	0.147 (0.619)
* I*/*αI*	15.56 (4.98)
* *Completeness (%)	99.9 (99.9)
* *Redundancy	11.9 (10.9)
	
*Refinement*	
* *Resolution (Å)	30-3.1 (3.2–3.1)
* *No. reflections	34,453
* R*_work/_*R*_free_	18.0/26.8
	
No. atoms	
* *Protein	13,596
* *Ligand dUMP	186
* *Ligand CBK115334	96
* *Water	11
	
B-factors	
* *Protein	45.3
* *Ligand dUMP	45.1
* *Ligand CBK115334	51.9
* *Water	30.9
	
R.m.s. deviations	
* *Bond lengths (Å)	0.014
* *Bond angles (°)	1.096

^*^Highest resolution shell is shown in parenthesis.

**Table 2:** Monitored LC-MS/MS transitions.

**Compound/Metabolite**	**Parent and daughter ions**
5-FU	128.8>41.9
FdU	244.9>155.0
FdUMP	324.9>195.0
FUR	260.9>171.0
EdU	250.9>135.8
EdUMP	330.9>195.0
TFT	249.9>179.7
TFTMP	374.9>179.4
Decitabine	227.0>93.8
Decitabine-MP	307.0>195.0
Pyrimethamine*	248.8>176.9
CBK115334*	284.9>188.9

^*^Analyzed with electrospray ionization in positive mode.
