# Supplementary material for: CETSA screening identifies known and novel thymidylate synthase inhibitors and slow intracellular activation of 5-fluorouracil
Source: Nat Commun. 2016 Mar 24;7:11040. doi: 10.1038/ncomms11040 (PMC4820820; doi:10.1038/ncomms11040)
Supplement: Supplementary Information — Supplementary Figures 1-17, Supplementary Tables 1-6, Supplementary Note 1 and Supplementary References [file ncomms11040-s1.pdf]

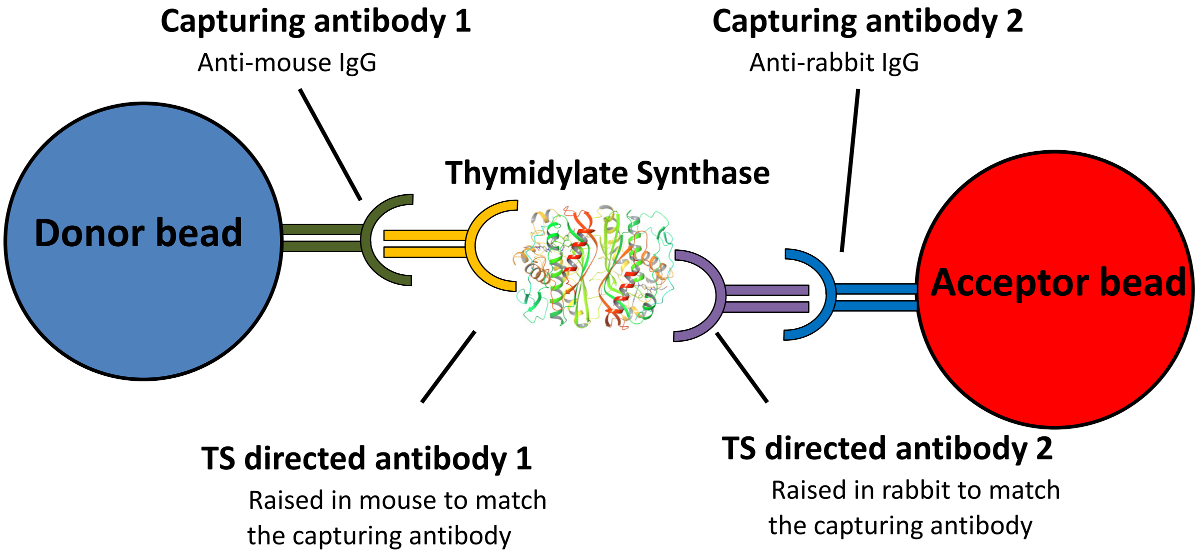

Supplementary Figure 1| Schematic outline of the assay principle for the AlphaScreen® assay used to detect human thymidylate synthase in cell lysates. Detection requires dual simultaneous recognition of native folded thymidylate synthase. This was achieved using antibodies from two different species, in this case from mouse (yellow) and rabbit (purple). Commercially available antibodies directed towards thymidylate synthase were efficiently screened using AlphaScreen® acceptor and donor beads functionalized with capturing antibodies recognizing the Fc domain of IgG antibodies from these two species (see Supplementary Table 1 for an overview of the antibodies included in this study). Singlet oxygen transfer between the donor and acceptor beads occurs only when the two bead types are close in space. This occurs when a productive complex involving the following components is formed in solution:

- Donor beads conjugated with an antibody recognizing the Fc domain of mouse derived antibodies
- Mouse derived antibody recognizing thymidylate synthase
- Thymidylate synthase
- Rabbit derived antibody recognizing thymidylate synthase
- Acceptor beads conjugate with an antibody recognizing the Fc domain of rabbit derived antibodies

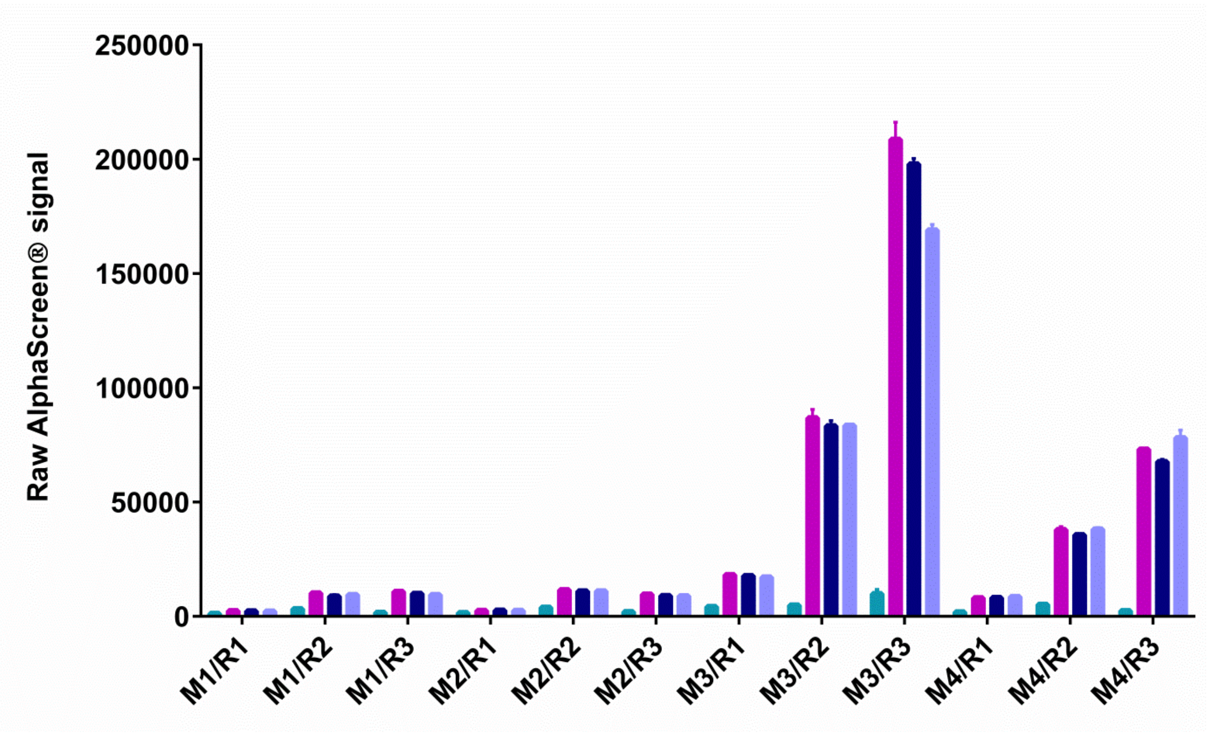

Supplementary Figure 2| Results from screening of antibody pairs for dual recognition of recombinant thymidylate synthase using AlphaScreen®. All possible combinations of four mouse-derived (M1-M4) and three rabbit-derived antibodies (R1-R3) recognizing different epitopes of thymidylate synthase (Supplementary Table 1) were tested. Four different conditions were included for each pair, with two of those being the absence (green) and presence of 2 nM of thymidylate synthase (magenta). Given our previous experience of ligand induced quenching of protein target recognition by the antibody pair (Jafari *et al.*, 2014) we also included a control containing 100 µM deoxyuridine monophosphate (dUMP) (blue) and a control containing 100 µM dUMP and 10 µM raltitrexed (lavender blue). Data are provided as the average and range from experiments done in duplicate at one independent test occasion. Four tentatively suitable pairs were identified and further testing was required for selection of the most appropriate pair.

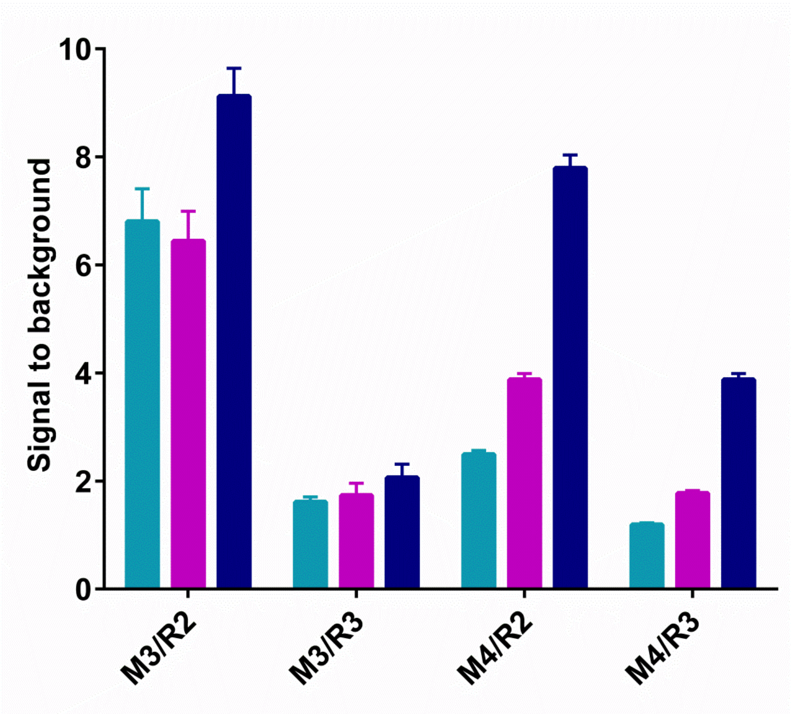

Supplementary Figure 3| Illustration of the kinetics for recognition of thymidylate synthase in cell lysates from K562 cells in the AlphaScreen® assay. Four different antibody combinations were selected for testing based on the results of the experiment in Supplementary Fig. 2. The experiment was performed to address the ability of the four different antibody pairs to recognize thymidylate synthase also in the context of cell lysates. Establishment of an equilibrium signal that remains stable over several hours is essential to allow parallel processing of multiple plates in screening campaigns. For each tested pair the signal to background is given as a function of time after addition of antibodies and beads: 2h (green), 6h (magenta) and 18h (blue). Data are provided as the average and standard error of mean from experiments done in triplicate at one test occasion. Based on the combination of an excellent signal to background and a comparatively fast and stable recognition of thymidylate synthase also in the context of the cell lysates we decided on a pair based on the mouse M3 and the rabbit R2 antibodies (Supplementary Table 1) for further assay development purposes.

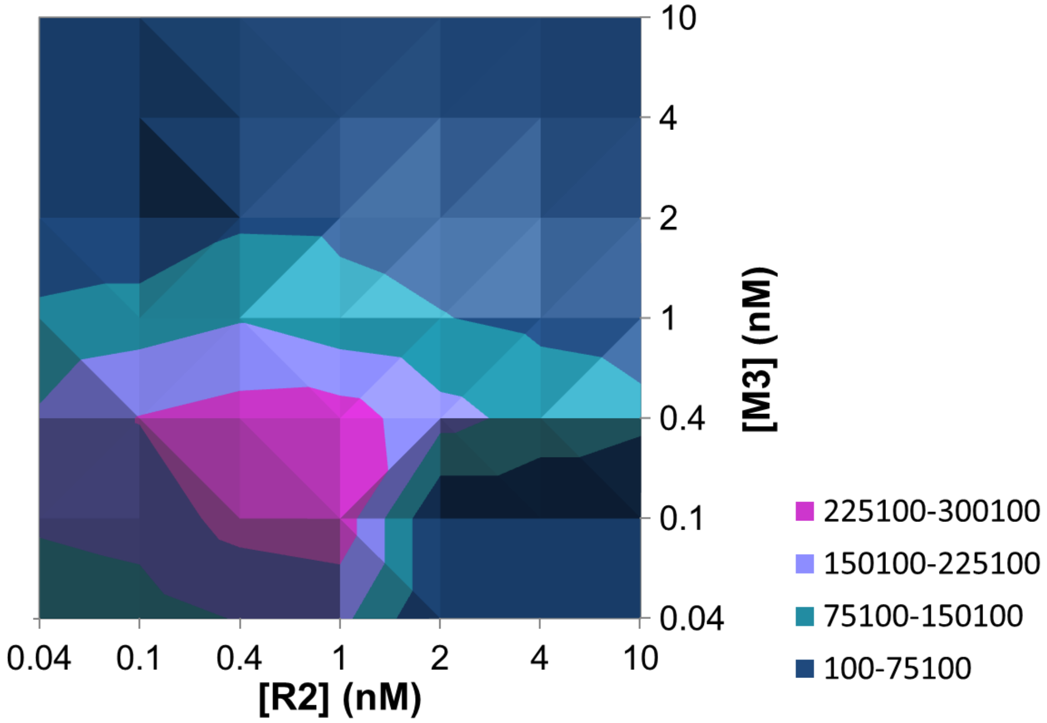

Supplementary Figure 4| Illustration of the AlphaScreen® response for thymidylate synthase as a function of antibody concentrations. Given that a multi-component complex must be formed to yield a productive assay signal (see Supplementary Fig. 1 for an illustration of the assay principle) the antibody concentrations must be titrated to avoid hook effects in the assay. Data are shown as a 3D contour graph where the concentrations of the antibodies directed towards thymidylate synthase are varied on the x-axis (R2) and y-axis (M3), respectively (see Supplementary Table 1 for details on the antibodies). The experiment was done based on a cell lysate from 7.5 million K562 cells per mL. Data are provided as the average from experiments done with n=2-4 for each condition obtained at one test occasion. Based on these results we decided to use 0.4 nM M3 and 1.0 nM R2 in the final screening assay.

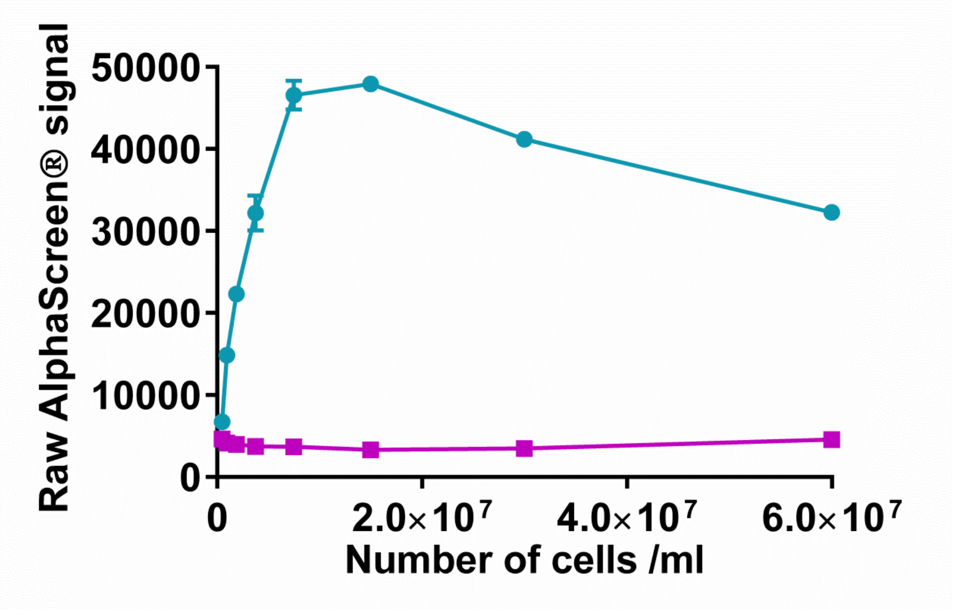

Supplementary Figure 5| Illustration of the AlphaScreen® signal for human thymidylate synthase as a function of K562 cell numbers. For assays measuring the content of soluble protein it is important to work in a range where the signal responds linearly with the cell numbers. Data are shown as the average and standard error of mean from triplicate samples obtained at one test occasion. Labels are as follows: cell lysate from cells kept at room temperature (green circle) or after heating to 52°C for 3 minutes (magenta square). This transient heat treatment denatures and precipitates thymidylate synthase in these cells (Fig. 1b in main text). In order to maximize the signal window without the risk of saturating the detection system we decided to use 5 million K562 cells per ml in the final screening assay.

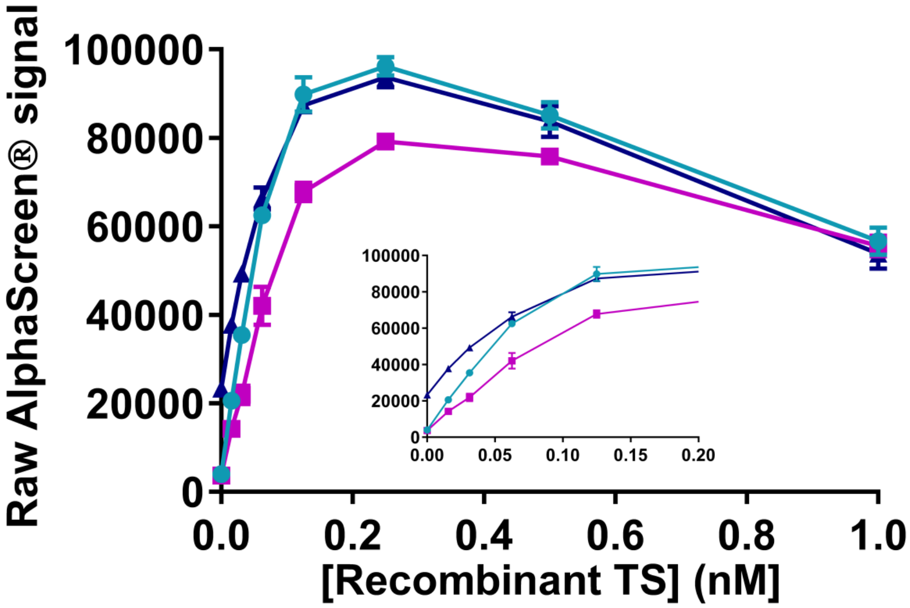

Supplementary Figure 6| Illustration of the change in AlphaScreen® signal as a function of added recombinant thymidylate synthase to K562 cell lysates. To address the specificity of the selected dual antibody pair (M3 and R2; Supplementary Table 1) for recognition of human thymidylate synthase in the context of a complex cell lysate we performed seeding experiments with thymidylate synthase added to unheated cell lysates (blue triangle), lysates heated to 52˚C for 3 minutes (magenta square), or to cell medium without any cells (green circle). The insert illustrates the same data, but restricted to the 0-0.2 nM concentration range. Data are shown from one test occasion as the average and standard error of mean (S.E.M) from triplicate samples. To stay within the linear range of the assay response the cell lysate background corresponded to 1 million K562 cells per ml. Although the background levels in the absence of TS are different for the unheated lysate, reflecting the TS levels in the K562 cells, there is a corresponding increase in the signal in both lysates, whereas the signal increase is somewhat steeper in the absence of the cell lysate background. Based on these data we conclude that the signal scales with thymidylate synthase content also in the cell lysates.

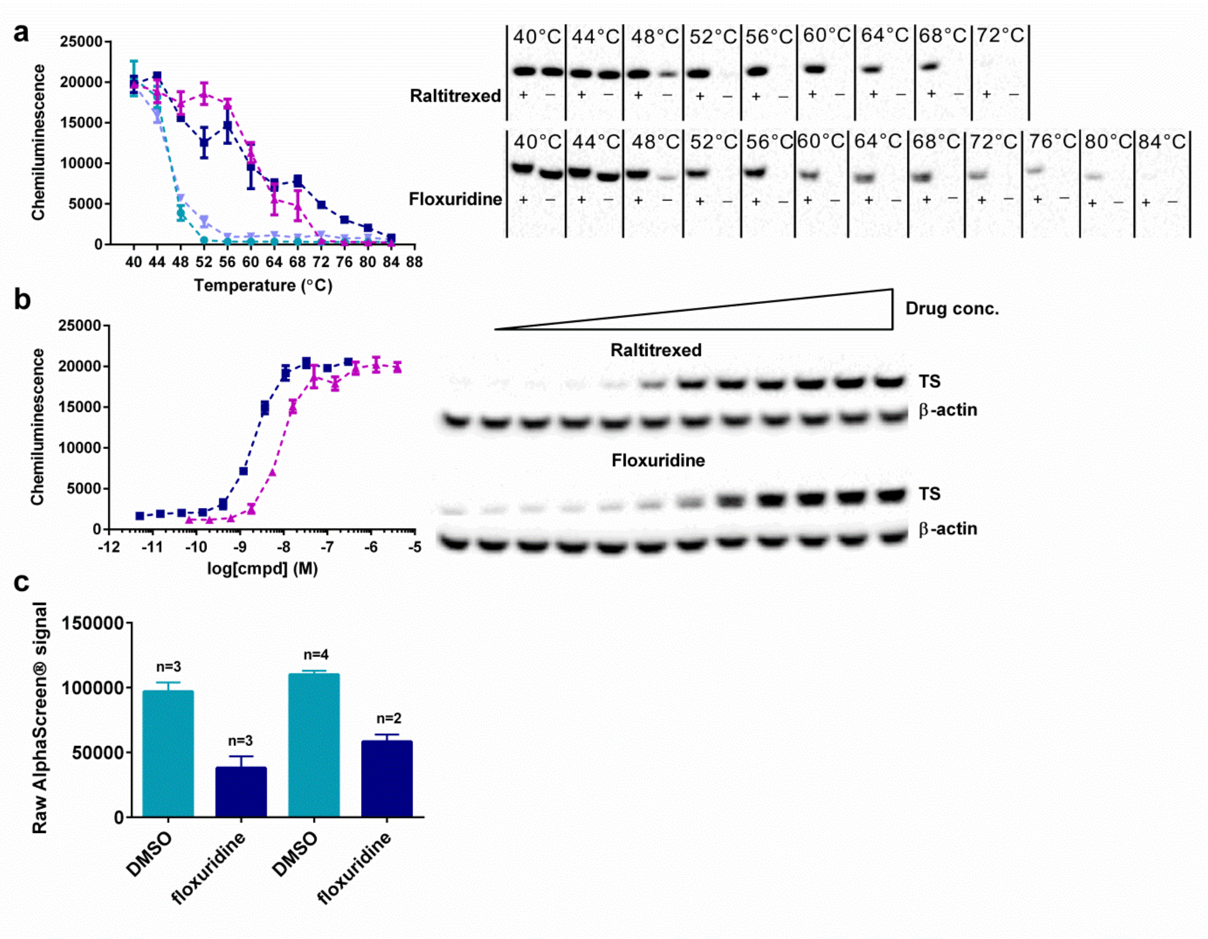

Supplementary Figure 7| Comparison of Western blot and AlphaScreen® based responses for floxuridine and raltitrexed. (**a**) Western blot data from CETSA experiments performed to determine T_{agg} curves for thymidylate synthase in live cells following a 2h preincubation period in the presence of DMSO (green circle and lavender blue downwards triangle) and 5 µM floxuridine (blue square) or 5 µM raltitrexed (magenta upwards triangle). To the right of the graphs are the corresponding chemiluminescence data. (**b**) Western blot based $ITDRF_{CETSA}$ following a 2h preincubation period at 37°C at different concentrations of floxuridine (starting at 300 nM and serially diluted in 11 steps down to 5 pM) (blue square) and raltitrexed (starting at 4 µM and serially diluted down to 67 pM) (magenta upwards triangle) and after heating for 3 minutes at 50°C. To the right of the graphs are the corresponding chemiluminescence data probing TS as well as the corresponding β-actin levels. All Western blot experiments were performed on K562 cells at a minimum of two independent biological replicates. (**c**) Illustration of the AlphaScreen® response in lysates from K562 cells for DMSO (green bars) and floxuridine treated cells (blue bars). Data are shown as the average and S.E.M. for two independent biological replicates following a 2h pre-incubation period. While Western blot experiments demonstrate similar protein levels between controls and treated samples (see panel **a**), we consistently observed a significant attenuation (~50%) of the AlphaScreen® signal for fluoropyrimidines. Intracellular enzymatic conversions of the fluoropyrimidines generate the active species FdUMP that forms a ternary covalent complex with thymidylate synthase and 5,10-methylenetetrahydrofolate (see panel

a for a characteristic shift in electrophoretic mobility of the ternary complex)[1]. As previously reported[2], we commonly observe ligand induced attenuation of the AlphaScreen signal® in the no-wash assay setup because the ligand is present also during detection. Despite the observation of signal attenuation for a small number of covalently binding compounds in this case we proceeded with the selected antibody pair and assay setup since it resulted in excellent assay performance.

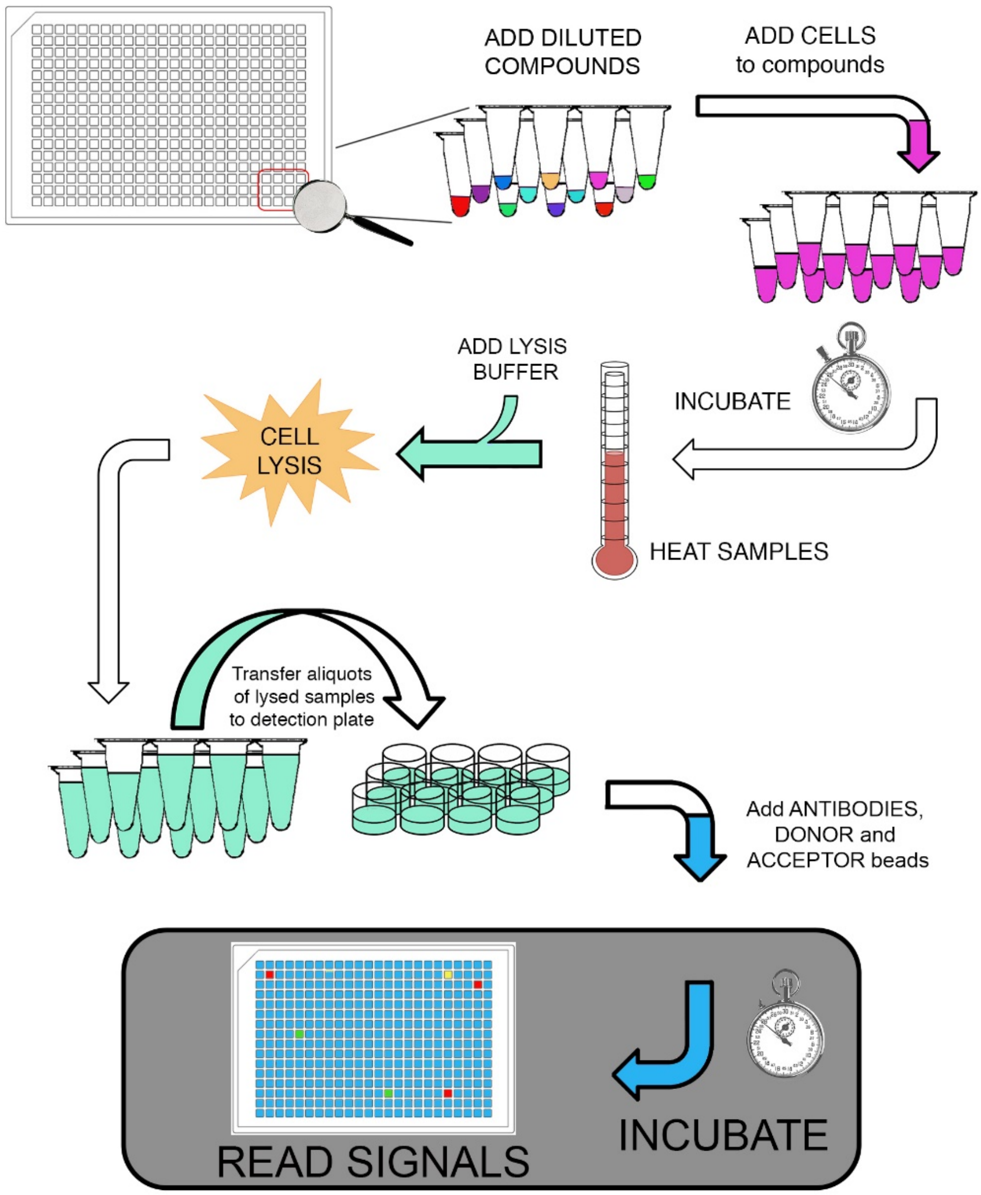

Supplementary Figure 8| Schematic outline of the small molecule screening logistics applied in the screening campaign on human thymidylate synthase using CETSA. Compounds and controls were dispensed to round-bottomed 384-well plates using an Echo 550 and diluted with supplemented cell medium using a Multidrop Combi. The diluted compounds were next transferred to 384-well PCR plates using a Bravo liquid handling station. K562 suspension cells were then added using a multipipette followed by preincubation for 2h in a cell incubator at 37°C. The PCR plates were next heated transiently to 50°C for three minutes followed by a controlled cooling to 20°C using a Roche Lightcycler480. The plates were then centrifuged briefly and subsequently cell lysis was accomplished by addition of lysis buffer using a Flexdrop IV. To ensure sufficient lysis the cell lysates were thoroughly mixed by ten repetitions of aspirations and dispenses using the 384-well head of the Bravo. Part of the lysates were then transferred to white 384-well plates followed by the addition of antibodies and AlphaScreen® acceptor and donor beads using a Multidrop Combi under subdued light. The plates were incubated over night at room temperature prior to detection in an Envision plate reader. As described in the Methods section the plates were sealed under several of these steps.

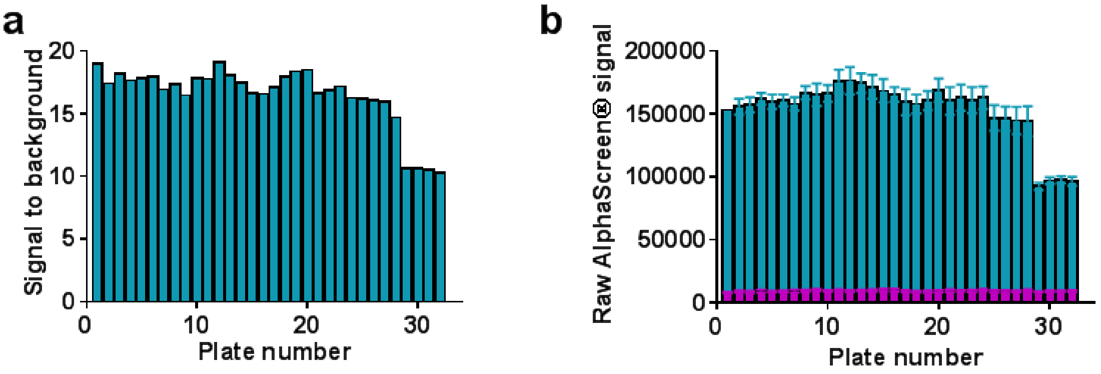

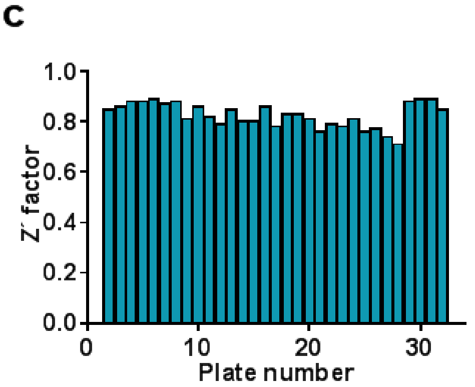

d

Hit Distribution within the plate

Row/Column	1	2	3	4	5	6	7	8	9	10	11	12	13	14	15	16	17	18	19	20	21	22	Row-wise:
A	0	0	0	0	0	0	0	0	0	0	0	0	0	0	0	0	0	0	0	0	0	0	0
B	0	1	0	0	0	0	0	0	0	0	0	0	0	0	0	0	1	0	0	0	0	0	2
C	0	0	0	0	0	0	0	0	0	0	0	0	0	1	0	0	1	0	0	0	0	0	2
D	1	0	0	0	0	0	1	1	0	0	1	0	1	0	1	0	1	0	0	0	0	0	7
E	0	0	0	0	0	0	0	0	0	1	0	0	0	0	0	0	0	0	0	0	0	0	1
F	0	0	0	1	0	0	0	0	0	0	1	0	0	1	0	0	1	0	1	0	0	0	5
G	0	0	1	0	0	0	0	0	1	1	1	0	0	0	1	0	0	0	0	0	0	0	5
H	0	0	0	1	0	0	0	0	0	0	0	0	1	1	0	0	0	0	1	0	0	0	4
I	0	0	0	0	0	1	0	0	0	0	0	0	0	0	0	0	0	0	0	0	0	1	2
J	1	0	0	0	1	1	0	0	0	0	0	0	0	0	0	0	0	0	0	0	1	0	4
K	0	0	0	0	0	2	1	0	0	0	0	2	0	0	0	0	0	0	0	0	0	0	5
L	0	1	0	0	1	0	0	0	0	1	0	2	1	0	0	1	0	0	1	0	0	0	8
M	0	0	1	0	0	0	0	1	0	0	0	1	2	0	0	0	1	0	0	0	0	0	6
N	0	0	1	0	0	0	0	1	0	0	0	1	1	0	0	0	0	0	0	0	0	0	4
O	0	0	0	0	0	0	0	0	0	0	1	0	1	1	1	0	0	0	0	0	0	0	4
P	0	1	0	1	0	0	0	0	1	0	0	0	1	1	0	0	0	0	0	0	0	1	6
Column-wise:	2	3	3	3	2	4	2	3	2	3	4	6	8	5	3	1	5	0	3	0	1	2	

Supplementary Figure 9| Illustration of the primary screening results using CETSA to measure target engagement of human thymidylate synthase in K562 cells. (**a**) Signal to background based on controls for each individual 384-well plate included in the screening campaign. The values were calculated based on averages of 16 each of negative (0.5% DMSO; column 23) and positive controls (100 nM raltitrexed; column 24). (**b**) Signal and background of the measured AlphaScreen® responses for the controls on each screening plate. Data are shown as the average and S.E.M. (**c**) Calculated Z´ factors based on negative and positive controls on each individual 384-well plate. (**d**) Illustration of the distribution of hits identified in the primary screen, *i.e.* the plate well location for solutions demonstrating more than 11.7% stabilization of human thymidylate synthase.

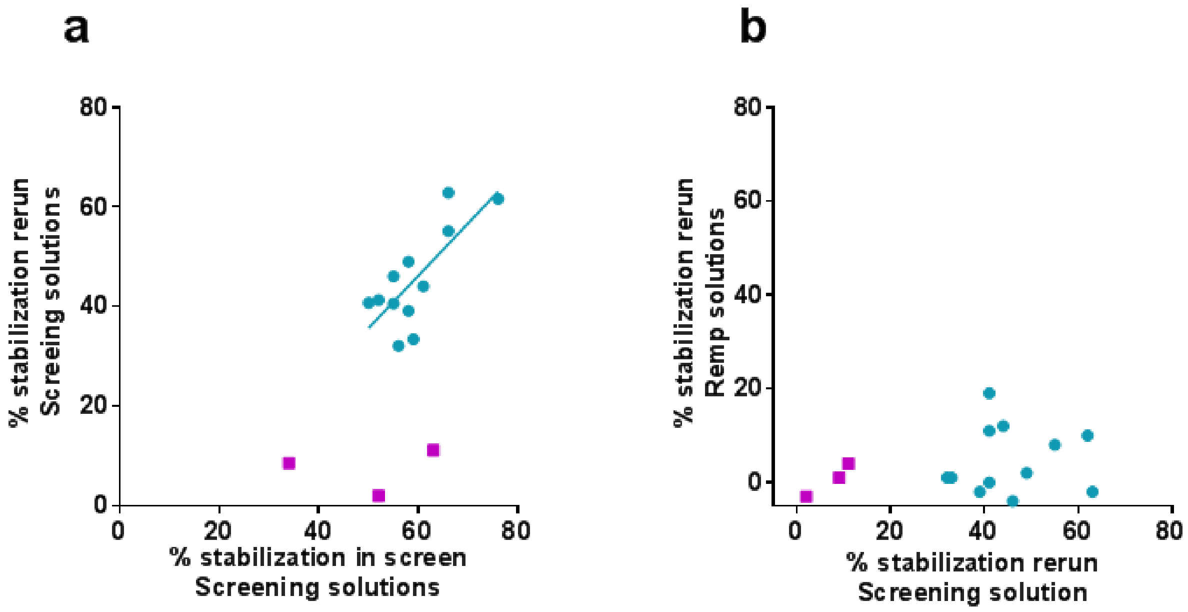

Supplementary Figure 10| Additional testing on compound solutions that did not reproduce activity in the first hit confirmation experiment. Several hits resulting in >30% apparent stabilization in the primary screen did not confirm activity when they were retested as a function of compound concentration (highlighted at the bottom of Supplementary Table 3). A key difference between these experiments was the use of different sources for the compound solutions. Whereas the screen was based on compound solutions plated from Labcyte 384 LDV plates, the hit confirmation experiment was based on compound solutions taken directly from a vial based storage system at CBCS. Additional experiments on these two sets of compound solutions were hence performed to investigate whether the CETSA screen was associated with a poor hit confirmation rate or whether there were different activities observed in the two sets of compound solutions. (**a**) Correlation between the responses observed in the primary screening campaign (single replicate) and a follow-up experiment based on solutions from the same Labcyte 384 LDV compound source plates (illustrated as the average of a duplicate obtained at one test occasion). Out of 15 tested solutions 12 reproduced significant activity (green circle) with a correlation coefficient of 0.56 between the two experiments, whereas three solutions failed to reproduce activity (magenta square). (**b**) Comparison of activities observed on solutions taken from the Labcyte 384 LDV compound source plates on the x-axis versus those prepared from the REMP vial storage system on the y-axis (in both cases illustrated as the averages of duplicate measurements obtained at one test occasion). Regardless of whether the hits reproducibly showed activity (green circle), or not (magenta square), when tested from the source plates that were applied in the primary screening campaign they failed to demonstrate activities approaching 30% stabilization when tested based on the REMP vial solutions. The source plates were generated by tip based transfer of solutions from the REMP-based vials to the compound

plates using a 96-well head and these solutions were then moved further with a 384-well head into 1536-well plates. In both these transfers the tips were washed extensively with DMSO between vials and plates. Despite these precautions careful inspection of the laboratory notes describing the generation of the Labcyte 384 LDV plates demonstrates that five of the contaminated solutions had been in contact with a pipette tip that had transferred a 10 mM floxuridine solution in a preceding step, while another three repeatedly active solutions were adjacent to wells containing floxuridine. Based on these observations we concluded that the original source plates are cross-contaminated and generated new plates for future screening campaigns.

a

This report was created by ACD/NMR Processor Academic Edition. For more information go to www.acdlabs.com/nmrproc/

2015-06-30 13:53:35

Acquisition Time (sec)	3.1654	*Comment*	Group LCBKI\ F12221080 KI1H DMSO {C:\Bruker\Topspin218} mahara 23				
Date	29 Jun 2015 16:45:20			*Date Stamp*	29 Jun 2015 16:45:20		
File Name	\\130.229.43.168\RDS_local\Agneta\data\mahara\nmr\1506cc074\10\pdata\1\1r					*Frequency (MHz)*	400.13
Nucleus	1H	*Number of Transients*	16	*Origin*	spect	*Original Points Count*	32768
Owner	nmr	*Points Count*	65536	*Pulse Sequence*	zg	*Receiver Gain*	181.00
SW(cyclical) (Hz)	10351.97	*Solvent*	DMSO-d6	*Spectrum Offset (Hz)*	4001.2981	*Spectrum Type*	STANDARD
Sweep Width (Hz)	10351.81	*Temperature (degree C)*	23.300				

^{1}H NMR (400 MHz, DMSO-d_6) δ ppm 2.58 (d, J=0.95 Hz, 3 H) 6.19 (s, 1 H) 7.48 - 7.59 (m, 3 H) 7.61 - 7.73 (m, 2 H) 7.98 (br. s., 2 H) 12.44 (s, 1 H)

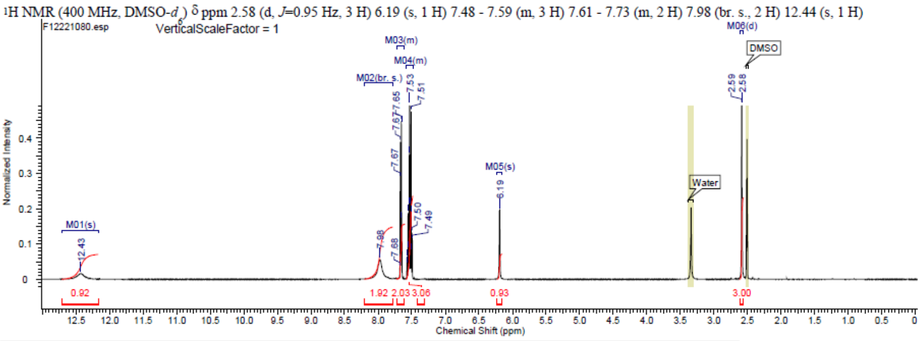

No.	(ppm)	Annotation	Layer No.	Created By	Created At	Modified By	Modified At
1	[2.49 .. 2.53]	DMSO	1	martin.haraldsso	ti 2015-06-30 08:02:45		
2	[3.31 .. 3.38]	Water	1	martin.haraldsso	ti 2015-06-30 08:02:45		

No.	Shift1 (ppm	H's	Type	J (Hz)	Multiplet1	(ppm)
1	2.58	3	d	0.95	M06	[2.56 .. 2.61]
2	6.19	1	s	-	M05	[6.16 .. 6.23]
3	7.53	3	m	-	M04	[7.48 .. 7.59]
4	7.66	2	m	-	M03	[7.61 .. 7.73]
5	7.98	2	br. s.	-	M02	[7.79 .. 8.20]
6	12.44	1	s	-	M01	[12.17 .. 12.71]

No.	(ppm)	Value	Absolute Value	Non-Negative Value
1	[2.5594 .. 2.61	3.00028062	8.26679552e+8	3.00028062
2	[6.1570 .. 6.22	0.92834902	2.55791792e+8	0.92834902
3	[7.4791 .. 7.58	3.05773425	8.42509952e+8	3.05773425
4	[7.6074 .. 7.72	2.02819014	5.58835520e+8	2.02819014
5	[7.7869 .. 8.20	1.92180657	5.29523200e+8	1.92180657
6	[12.1714 .. 12.7	0.92178243	2.53982464e+8	0.92178243

b

Data File C:\HPCHEM\1\DATA\1506RG\1143.D Sample Name: CBK115334

F12221080

```
=====================================================================
Injection Date  : 6/30/2015 8:31:09 AM          Seq. Line :   2
Sample Name     : CBK115334                      Location : Vial 4
Acq. Operator   :                                     Inj :   1
Acq. Instrument : Instrument 1                 Inj Volume : Inj prog
Sequence File   : C:\HPCHEM\1\SEQUENCE\USETHIS.S
Method          : C:\HPCHEM\1\METHODS\STANDARD\SX1097X3.M
Last changed    : 10/17/2013 1:29:39 PM by Doris_rg
STANDARD METHOD FOR REGISTRATION INTO CHEMSPEC
XTerra C18 50x3.0 mm, 10-97% acetonitrile in 3 min, 1 ml/min
215-395 & 254 nm, A=10mM NH4HCO3 (pH10), B=MeCN
```

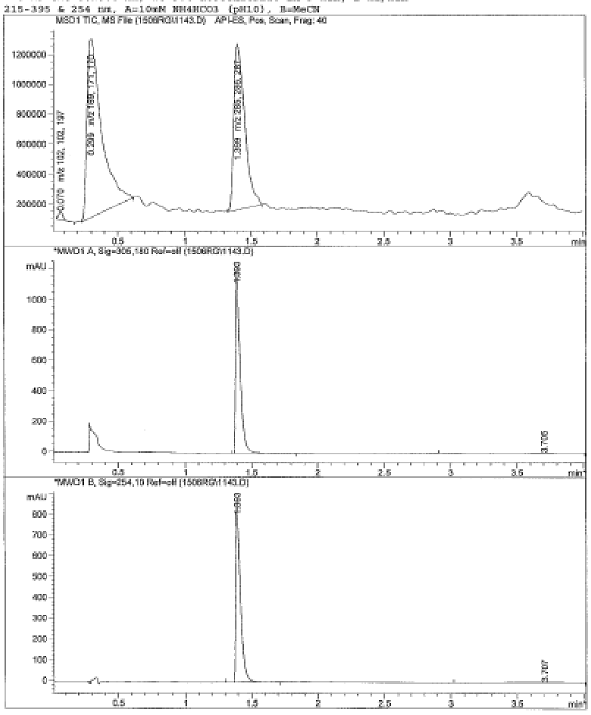

c

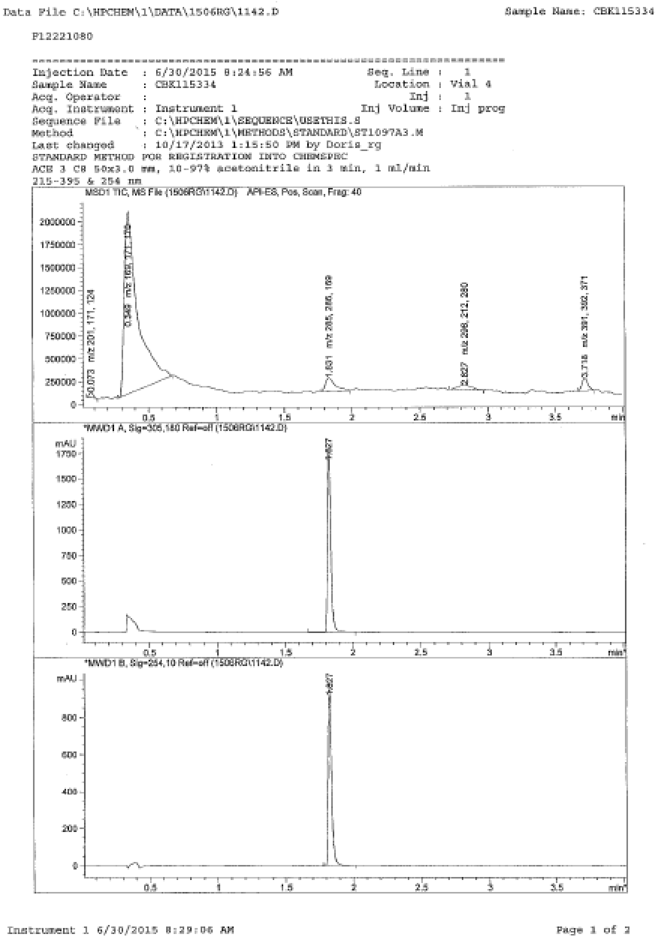

Supplementary Figure 11| Characterization of the novel thymidylate synthase inhibitor CBK115334 using nuclear magnetic resonance (NMR) spectroscopy and high-pressure liquid chromatography (HPLC). (**a**) ^{1}H NMR spectra of CBK115334 in deuterated DMSO using a Bruker DPX400 spectrometer (400 MHz). Chemical shifts are as follows: ^{1}H NMR (400 MHz, DMSO-d_6) δ ppm 2.58 (d, J=0.95 Hz, 3 H) 6.19 (s, 1 H) 7.48 - 7.59 (m, 3 H) 7.61 - 7.73 (m, 2 H) 7.98 (br. s., 2 H) 12.17 - 12.71 (s, 1 H). ^{1}H-NMR confirmed the expected compound. (**b**) Results from HPLC−MS analysis on an Agilent/HP 1200 system 6110 mass spectrometer with electrospray ionization (ESI+). HPLC−MS methods were as follows. Method 1: Waters XBridge C18 3.5 μm column (3.0 mm × 50 mm), 3.5 min gradient mobile phase [CH_3CN] / [10 mM NH_4HCO_3/H_2O]. (**c**) Method 2: ACE C8 3 μm column (3.0 mm × 50 mm), mobile phase [0.1% TFA/CH_3CN] / [0.1% TFA/H_2O]. Absorbance was monitored at 305 ± 90 and 254 nm. All solvents used were HPLC grade. HPLC-MS confirmed the correct molecular mass and showed a pure compound. The compound is commercially available and previously known from the literature[3,4].

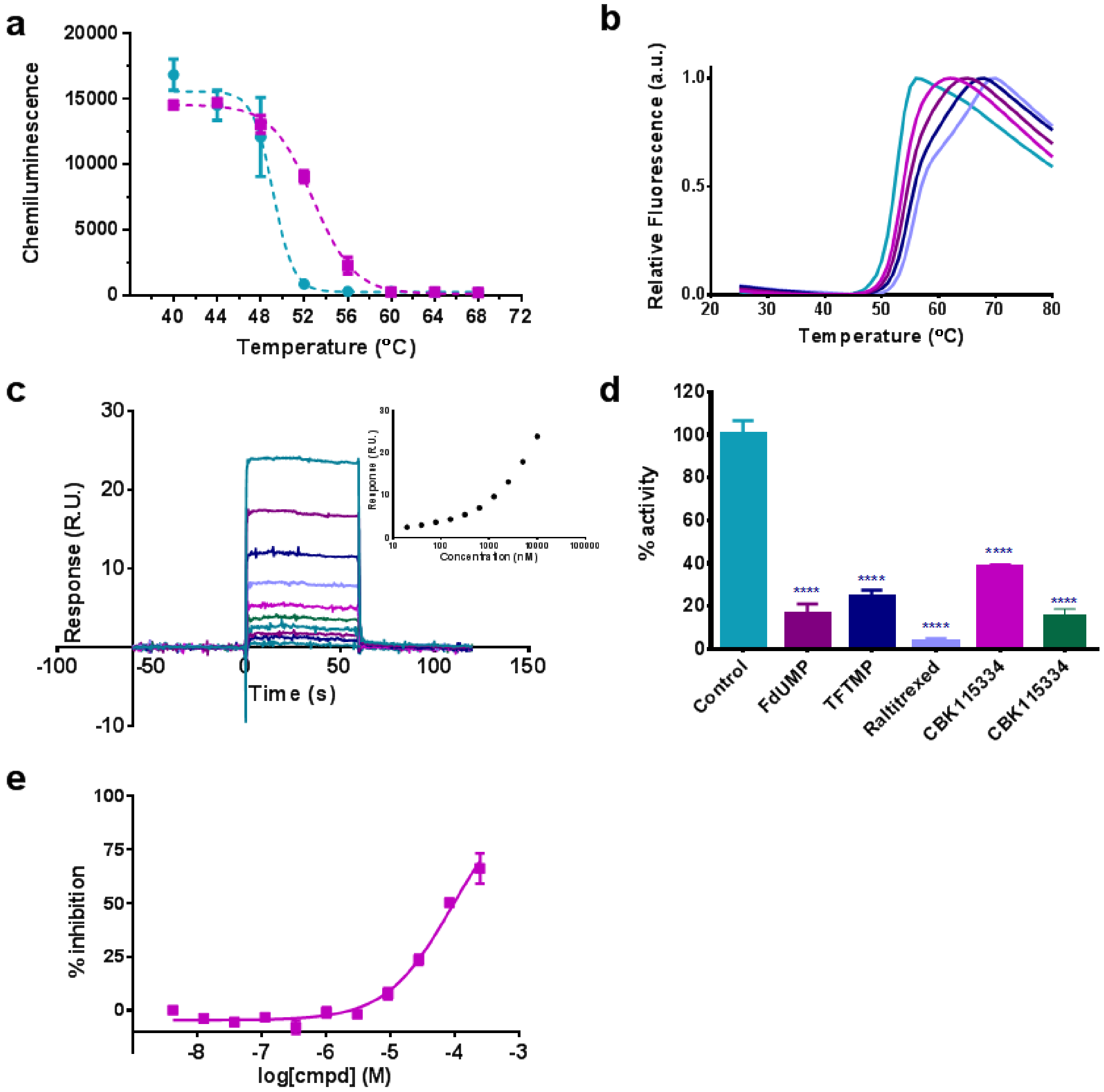

Supplementary Figure 12| Thermal shift, surface plasmon resonance, in lysate CETSA and cell viability data for CBK115334. (**a**) Western blot based CETSA derived T_{agg} curves for TS in K562 cell lysates in the presence of 200 μM CBK115334 (magenta square) or DMSO (green circle). All experiments were performed on K562 cells using Western blots at a minimum of three independent replicates. (**b**) Thermal shift *in vitro* assay of CBK115334 in the presence of 500 μM dUMP and DMSO (green), 12.5 μM (magenta), 25 μM (purple), 50 μM (blue) and 100 μM (lavender blue) CBK115334, respectively. (**c**) Sensorgrams and steady state affinity data from surface plasmon resonance experiments investigating the binding of CBK115334 to recombinant human thymidylate synthase in the presence of 200 μM dUMP. The compound concentrations used were 10 μM, 5 μM, 2.5 μM, 1.25 μM, 625 nM, 312 nM, 156 nM, 78 nM, 39 nM and 20 nM (from top to bottom in the graph). The steady state data are shown in the insert. These data demonstrate binding to thymidylate synthase in the low μM range, but given that the signal does not saturate it is not possible to get an accurate estimate of the affinity. (**d**) TS activity in the presence of DMSO (green), 10 μM

FdUMP (dark purple), 10 µM TFTMP (blue), 10 µM raltitrexed (lavender blue), 10 µM CBK115334 (light purple) and 100 µM CBK115334 (green), respectively. Data are presented as the average and S.E.M. of triplicate samples at one test occasion. (**e**) K562 cell viability measured following 72 hours incubation at 37°C and 5% CO_2 in the presence of different concentrations of CBK115334. All data were normalized based on DMSO and staurosporine controls to define complete inhibition. The solid line represents the best fit to a four-parameter dose-response model within GraphPad Prism resulting in a IC_{50} value of 88±15 µM. Data are presented as the average and S.E.M. of triplicate samples at one test occasion.

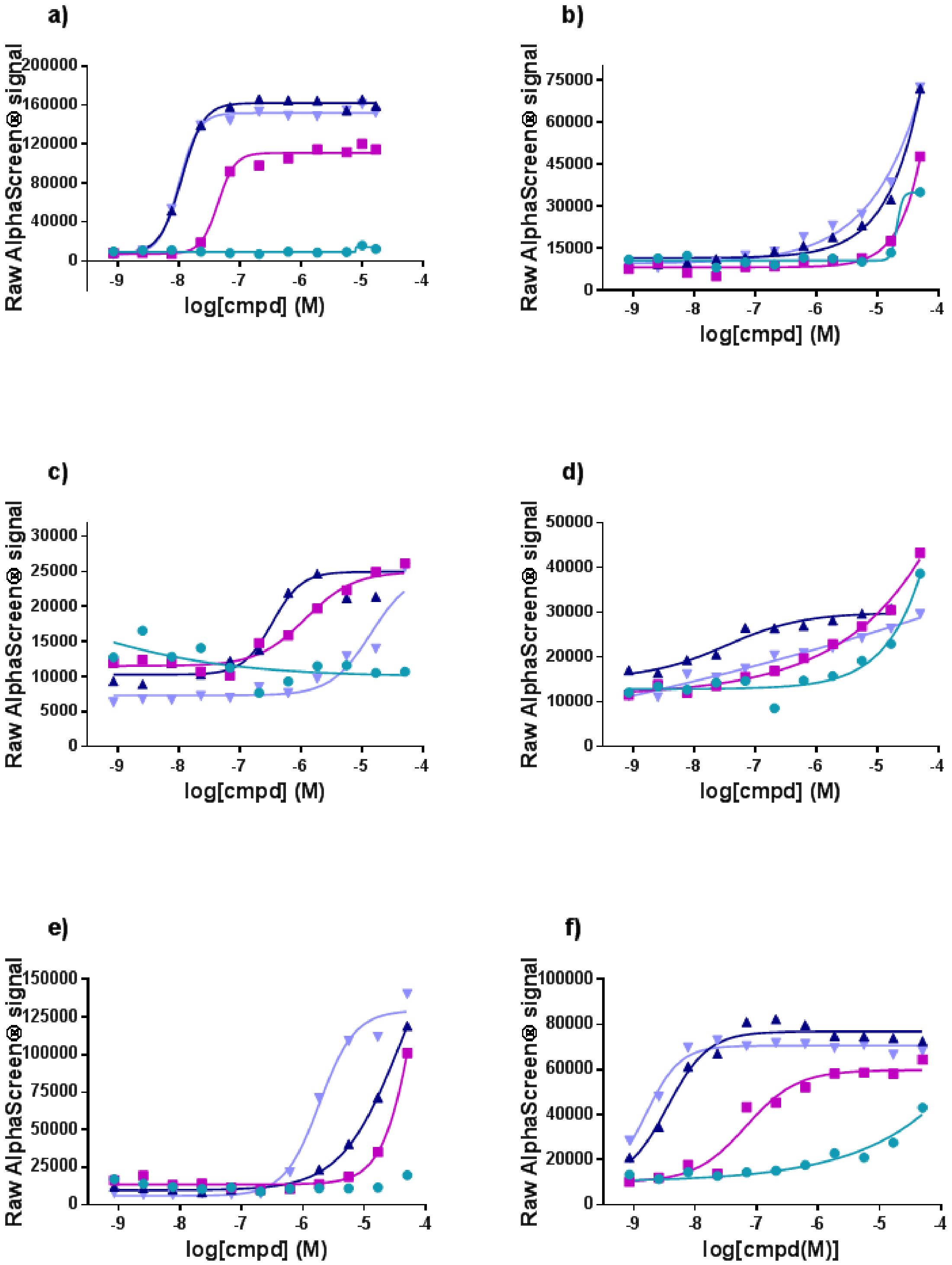
a)
Raw AlphaScreen® signal
200000
160000
120000
80000
40000
0
-9
-8
-7
-6
-5
-4
log[cmpd] (M)
b)
Raw AlphaScreen® signal
75000
60000
45000
30000
15000
0
-9
-8
-7
-6
-5
-4
log[cmpd] (M)
c)
Raw AlphaScreen® signal
30000
25000
20000
15000
10000
5000
0
-9
-8
-7
-6
-5
-4
log[cmpd] (M)
d)
Raw AlphaScreen® signal
50000
40000
30000
20000
10000
0
-9
-8
-7
-6
-5
-4
log[cmpd] (M)
e)
Raw AlphaScreen® signal
150000
125000
100000
75000
50000
25000
0
-9
-8
-7
-6
-5
-4
log[cmpd] (M)
f)
Raw AlphaScreen® signal
100000
80000
60000
40000
20000
0
-9
-8
-7
-6
-5
-4
log[cmpd(M)]

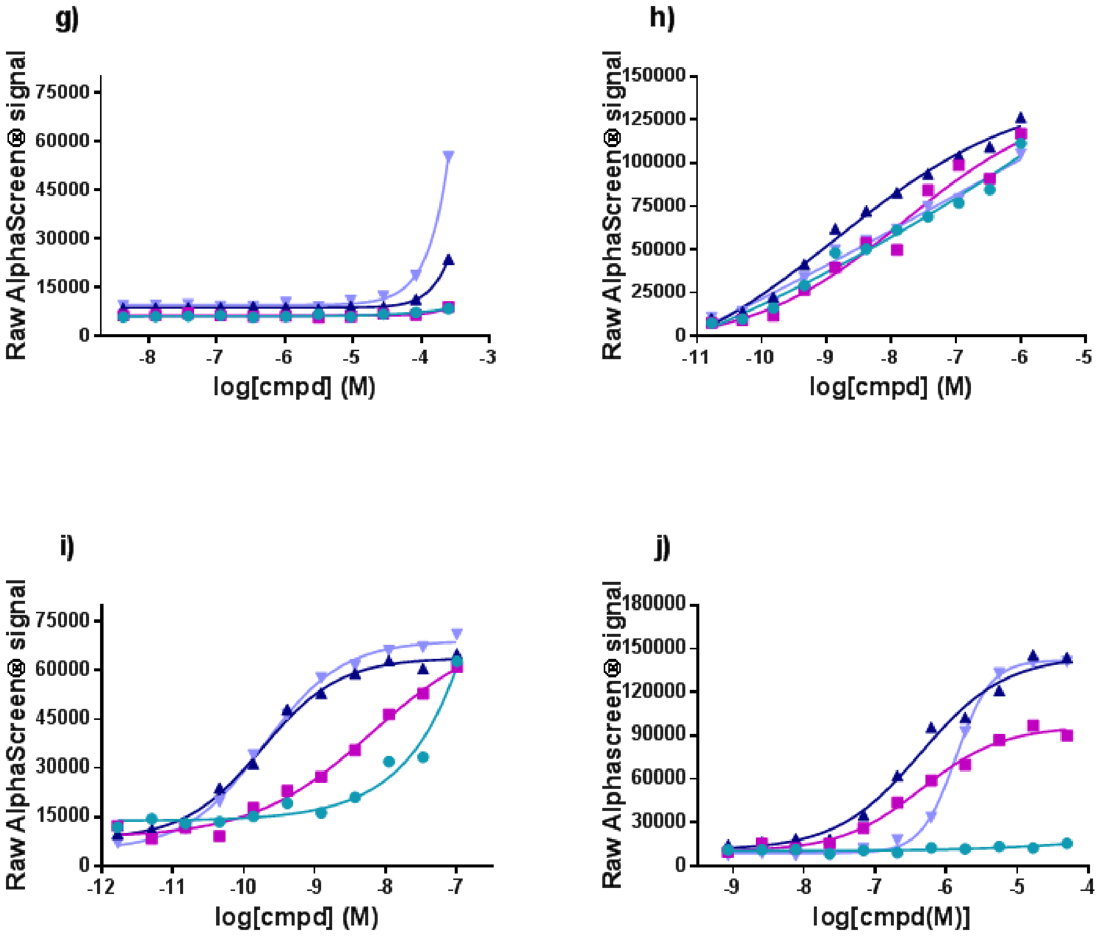

Supplementary Figure 13| Isothermal dose-response fingerprints using CETSA for selected hit compounds as a function of pre-incubation time in K562 cells. The $ITDRF_{CETSA}$ data were obtained by heating the cells transiently for 3 minutes at 50°C following various lengths of the pre-incubation time at a concentration of 5 million cells/ml. Symbols in each graph are as follows: 10 min (green circle), 30 min (magenta square), 2h (blue upwards triangle) and 6h(lavender blue downwards triangle). Data were obtained for methotrexate (**a**), CBK201253 (**b**), CBK201221 (**c**), CBK201230 (**d**), pyrimethamine (**e**), 5-fluorouridine (FUR), which was included in this effort although it was not part of the screen, (**f**), CBK115334 (**g**), 5-ethynyl-2´-deoxyuridine (EdU) (**h**), 5-trifluoro-2´-deoxythymidine (TFT) (**i**), and decitabine (**j**). The solid lines represent best fits to a saturation binding curve function resulting in half-maximal stabilization of thymidylate synthase. Representative data are provided as the average and S.E.M. from experiments done in quadruplicate at one test occasion. Some compounds were tested at two occasions as shown in Fig. 3 in the main text. Final concentrations of the assay reagents in the detection step was 1 nM rabbit polyclonal anti-TS IgG (15047-1-AP, Proteintech), 0.4 nM mouse monoclonal anti-TS IgG (sc-376161, Santa Cruz), 40 µg mL^{-1} AlphaScreen® anti-mouse donor beads and 10 µg mL^{-1} AlphaScreen® anti-rabbit acceptor beads.

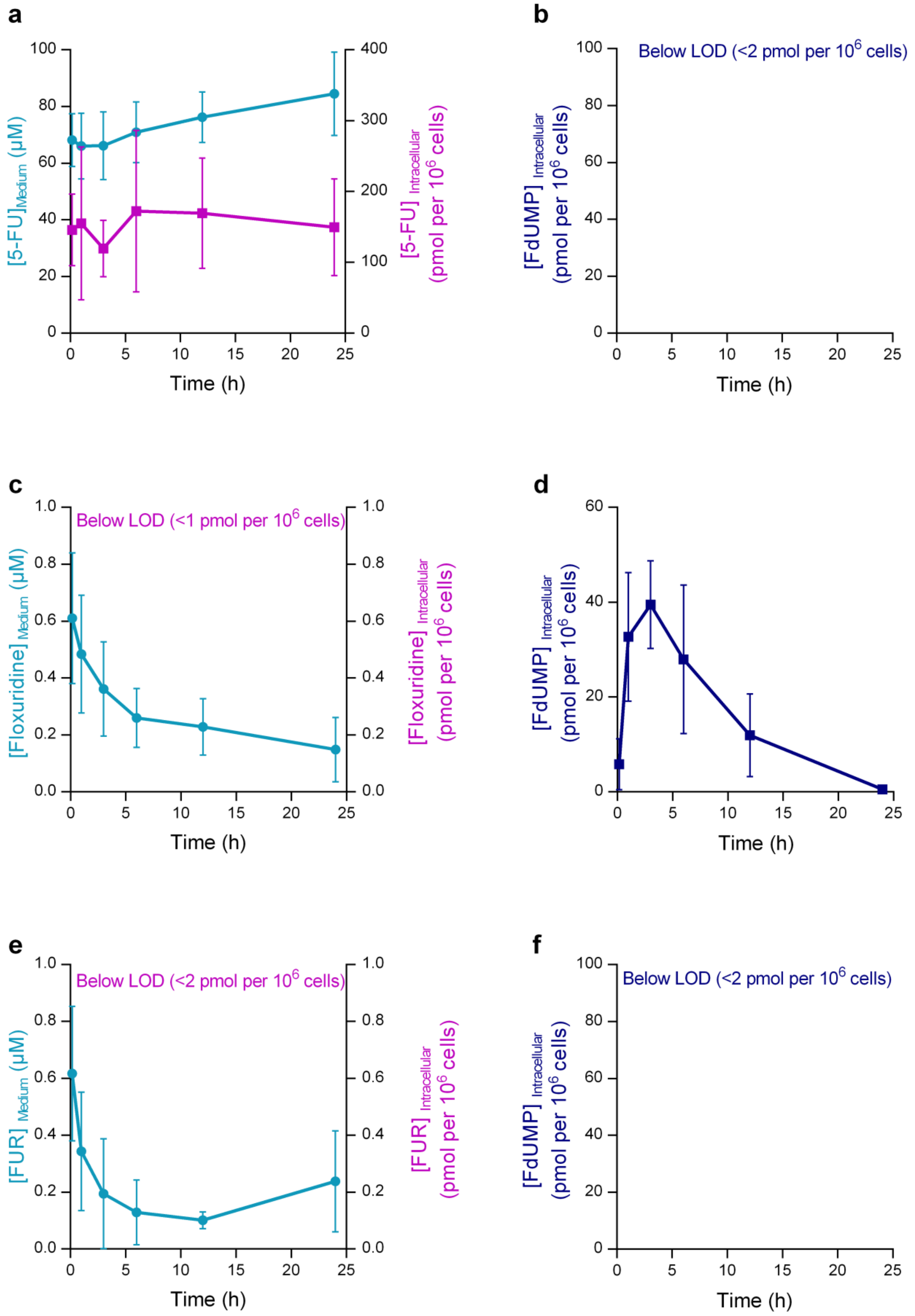
a
[5-FU]Medium (µM)
[5-FU] Intracellular (pmol per 10^6 cells)
Time (h)
b
Below LOD (<2 pmol per 10^6 cells)
[FdUMP] Intracellular (pmol per 10^6 cells)
Time (h)
c
Below LOD (<1 pmol per 10^6 cells)
[Floxuridine] Medium (µM)
[Floxuridine] Intracellular (pmol per 10^6 cells)
Time (h)
d
[FdUMP] Intracellular (pmol per 10^6 cells)
Time (h)
e
Below LOD (<2 pmol per 10^6 cells)
[FUR] Medium (µM)
[FUR] Intracellular (pmol per 10^6 cells)
Time (h)
f
Below LOD (<2 pmol per 10^6 cells)
[FdUMP] Intracellular (pmol per 10^6 cells)
Time (h)

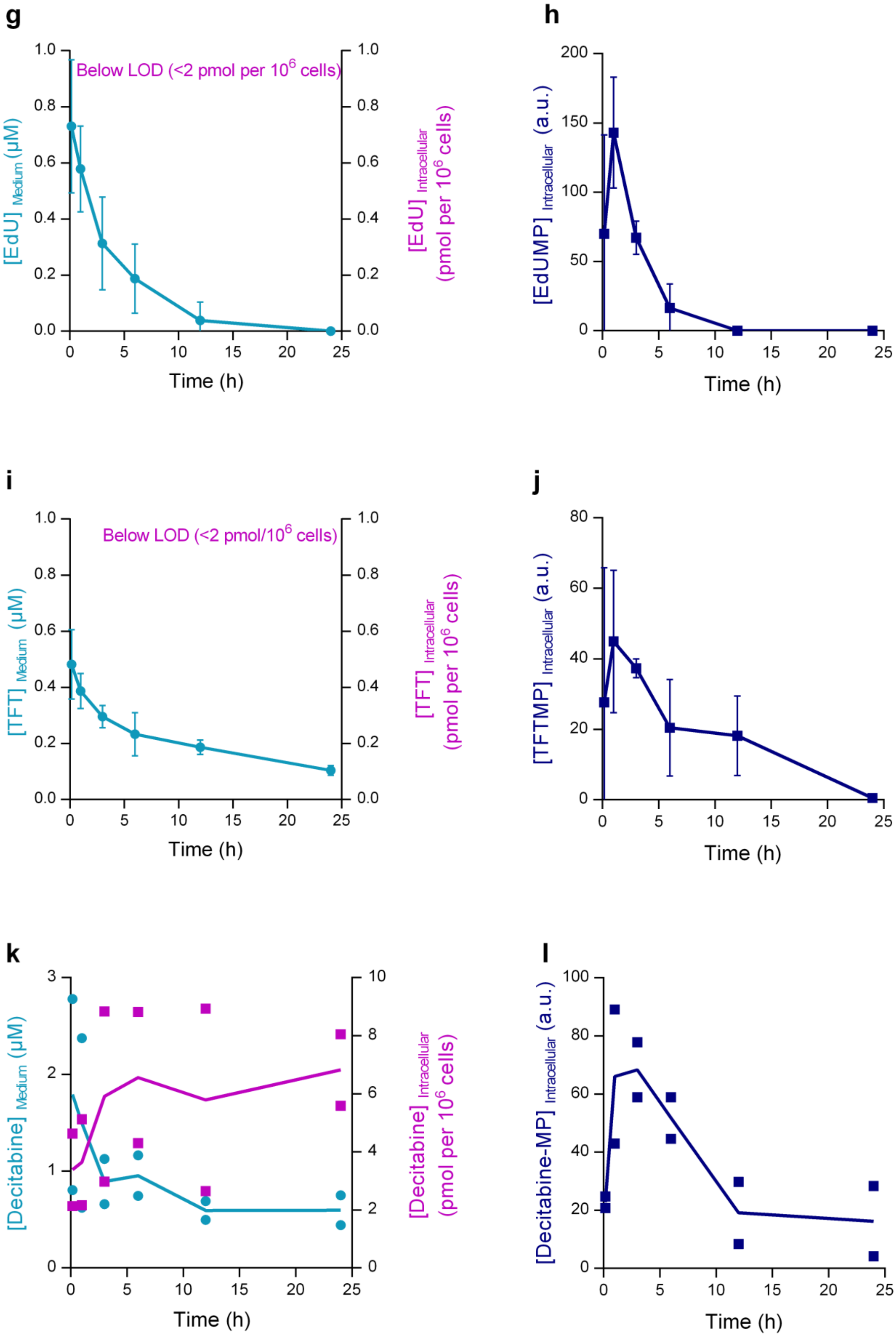

g
Below LOD (<2 pmol per 10^6 cells)
[EdU] Medium (μM)
[EdU] Intracellular (pmol per 10^6 cells)
Time (h)
h
[EdUMP] Intracellular (a.u.)
Time (h)
i
Below LOD (<2 pmol/10^6 cells)
[TFT] Medium (μM)
[TFT] Intracellular (pmol per 10^6 cells)
Time (h)
j
[TFTMP] Intracellular (a.u.)
Time (h)
k
[Decitabine] Medium (μM)
[Decitabine] Intracellular (pmol per 10^6 cells)
Time (h)
l
[Decitabine-MP] Intracellular (a.u.)
Time (h)

m

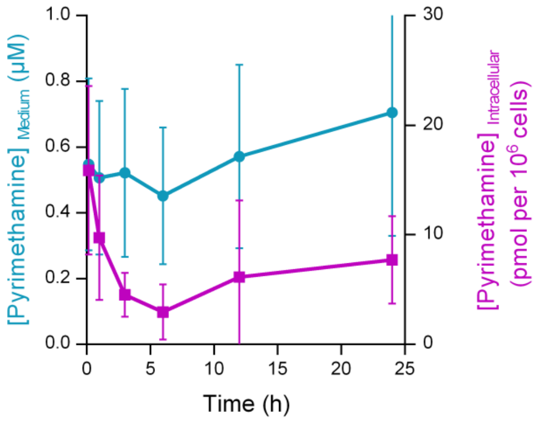

n

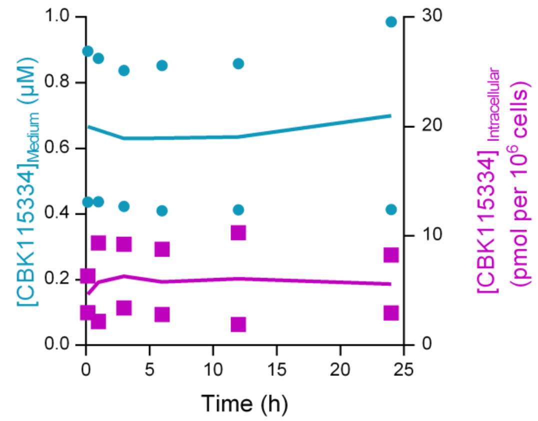

Supplementary Figure 14| Data on extracellular and intracellular concentrations of selected hit compounds using LC-MS. Extracellular (green circle) and intracellular concentrations of selected hit compounds (magenta square) and monophosphorylated metabolites (blue square) measured after various pre-incubation times with K562 cells using LC-MS/MS. (**a**,**b**) Incubation with 100 µM 5-FU. (**c**,**d**) Incubation with 1 µM floxuridine. (**e**,**f**) Incubation with 1 µM 5-fluorouridine (FUR) (included in this effort although it was not part of the screen). (**g**,**h**) Incubation with 1 µM EdU. (**i**,**j**) Incubation with 1 µM TFT. (**k**,**l**) Incubation with 1 µM decitabine. (**m**) Incubation with 1 µM pyrimethamine. (**n**) Incubation with 1 µM CBK115334.

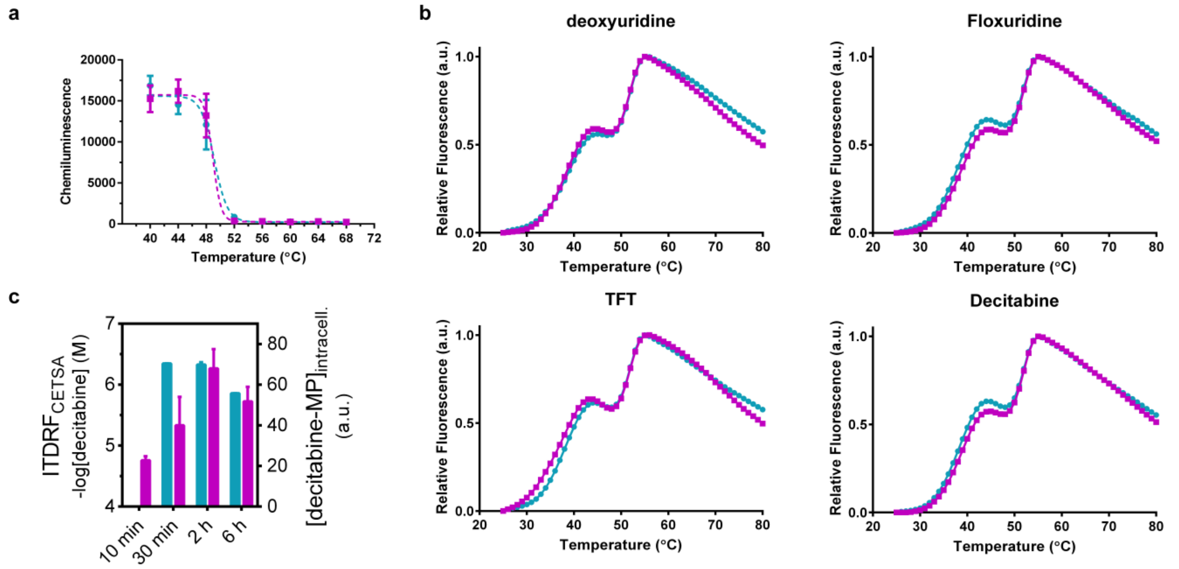

Supplementary Figure 15| Western blot based CETSA data on K562 lysates, thermal shift data on recombinant thymidylate synthase and time dependence of target engagement in K562 cells for decitabine. (**a**) T_{agg} curves for TS in the presence of 200 µM decitabine (magenta square) or DMSO (green circle). The experiment was performed on K562 cell lysates using Western blots at a minimum of three independent replicates. (**b**) Thermal shift *in vitro* assay for selected nucleosides (magenta) and controls (green). Apo-TS protein (control) has a bi-phasic melting profile. (**c**) Half-maximal stabilization of TS observed in CETSA experiments (green) and intracellular concentration of FdUMP (magenta) as a function of preincubation time with decitabine (see also Supplementary Figs. 13j and 14l for details). The CETSA data are presented as the average and range from two independent experiments. The LC-MS/MS data are provided as the average and S.E.M. from experiments done at three different occasions.

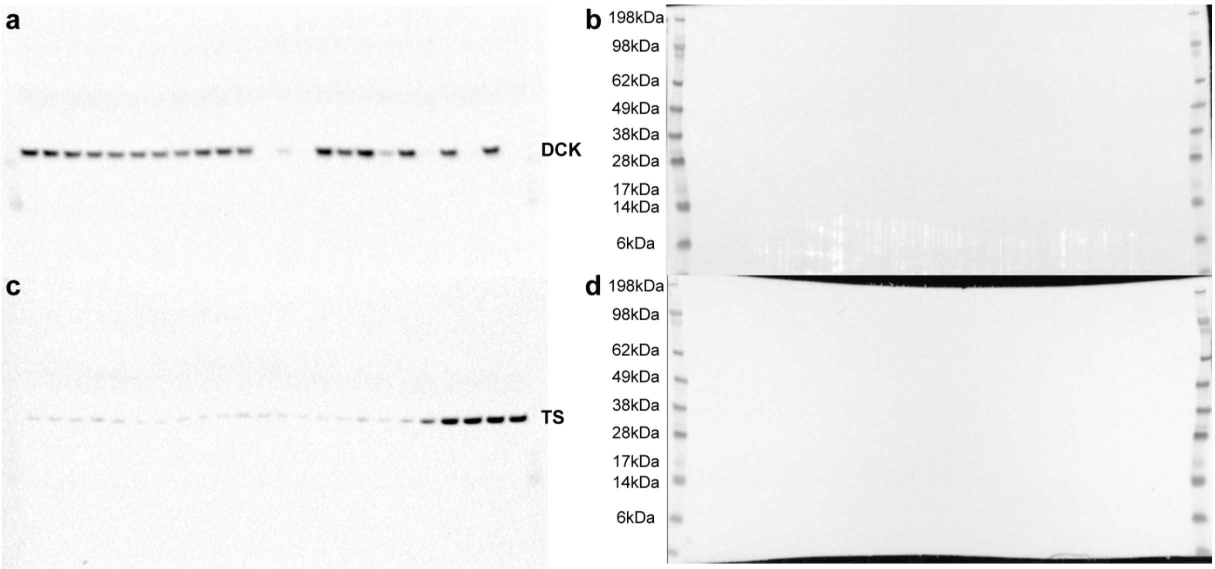

Supplementary Figure 16| Full blots from CETSA experiments on decitabine metabolism. (**a**) Chemiluminiscence blot probing DCK levels (corresponding to Fig. 4b in main text) (**b**) The corresponding molecular weight marker image. (**c**) Chemiluminiscence blot probing TS levels (corresponding to Fig. 4c in the main text). (**d**) The corresponding molecular weight marker image.

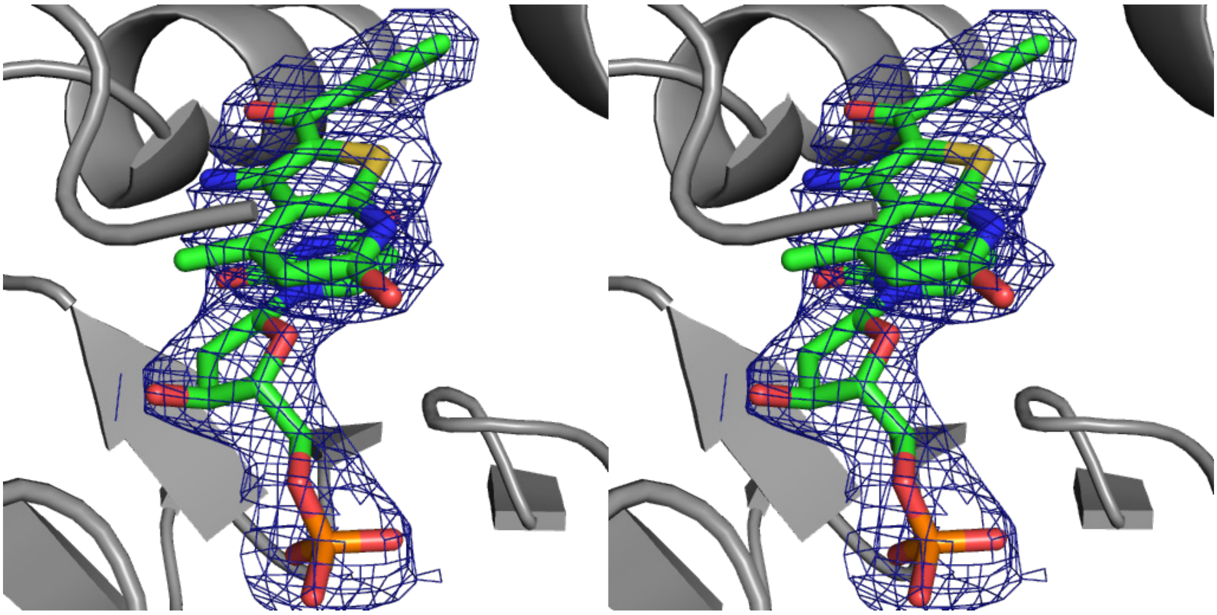

Supplementary Figure 17| Stereo image of the electron density map for dUMP and CBK115334 in the active site of thymidylate synthase. The 2Fo-Fc map around dUMP and CBK115334 is contoured in blue at 1.0 sigma level.

Supplementary Table 1| Rabbit and mouse derived antibodies directed towards human thymidylate synthase and tested in the AlphaScreen® assay

Antibody	Species	Isotype	Vendor	Product number	Recognized epitope	Vendor claimed validity for:
**M1**	Mouse	IgG1	Santa Cruz	sc-33679	Recombinant TS	WB, IP, IF, IHC(P) and FCM
**M2**	Mouse	IgG1	Santa Cruz	sc-56492	Recombinant TS	WB, IP, IF and FCM
**M3**	Mouse	IgG2b	Santa Cruz	sc-376161	Amino acids 99-127 within an internal region of TS	WB, IP, IF, IHC(P) and ELISA
**M4**	Mouse	IgG1κ	Sigma Aldrich	WH0007298M1	Amino acids 111-211	IHC, indirect ELISA, indirect IF, WB
**R1**	Rat		Avivasysbio	ARP48543_P050	C-terminal region	WB
**R2**	Rat	IgG	Proteintech	15047-1-AP	Recombinant TS	ELISA, WB, IHC, IF
**R3**	Rat	IgG	Cell signaling	D5B3	Residues near the C-terminus	WB, IF, IHC and FC

Supplementary Table 2| Small molecule screening information

Category	Parameter	Description
**Assay**	Type of assay	CETSA, Cellular Thermal Shift Assay with AlphaScreen® detection of target protein
	Target	Human thymidylate synthase; TS; dTMP synthase
	Primary measurement	Detection of remaining native TS after heat challenge in presence of test compounds.
	Key reagents	Anti-rabbit IgG (Fc specific) AlphaLISA® Acceptor Beads, PerkinElmer, AL104C<br>Anti-Mouse IgG Alpha Donor beads, PerkinElmer, AS104D<br>Anti TS mouse monoclonal IgG2b, Santa Cruz Biotechnology, sc-376161<br>Anti TS rabbit polyclonal, Proteintech, 15047-1-AP<br>AlphaLISA Immunoassay buffer 10X, PerkinElmer, AL000C<br>Surefire lysis buffer 5X, Perkin Elmer
	Assay protocol	See online methods and supplementary results.
	Additional comments	
**Library**	Library size	10,928 compounds
	Library composition	The library consists of a chemically diverse collection of compounds containing both commercial and internal compounds (donation from Biovitrum). The tested compounds includes also 1199 known bioactive molecules from the Prestwick chemical library and 192 nucleosides from Barry and Associates
	Source	The screen was done based on plating of 10 mM DMSO solutions from Labcyte 384 LDV plates using an Echo 550
	Additional comments	See Online Materials for further details on the composition of the CBCS primary screening set
**Screen**	Format	384-well format.<br>Compound plate: 384-well PP plate, Greiner, 784201<br>Assay plate: 384-well PCR plates from Bio-Rad, HSR-4805<br>Detection plate: 384-well Optiplate, Perkin Elmer, 6008280
	Concentration(s) tested	Compound concentration 50 µM, DMSO concentration 0.5%
	Plate controls	Positive control 100 nM Raltitrexed<br>Negative control 0.5% DMSO
	Reagent/ compound dispensing system	Compound dispensing system: Echo 550 from Labcyte<br>Reagent dispensing system: Multidrop combi from Thermo Scientific, FlexDrop IV from PerkinElmer and Bravo Automated Liquid Handling Platform from Agilent
	Detection instrument and software	Plate heating: Lightcycler 480 from Roche<br>Detection: Envision 2104 multilabel reader from PerkinElmer
	Assay validation/QC	Positive control: average 9308, standard deviation 932<br>Negative control: average 148051, standard deviation 28860<br>Average Z´ factor/plate: 0.83
	Correction factors	Not applicable
	Normalization	Data are normalized to the positive (100% stabilization) and negative controls (0% stabilization) on each plate and are expressed as % stabilization
	Additional comments	The screen was done at Chemical Biology Consortium Sweden at Karolinska institutet, Sweden
**Post-HTS analysis**	Hit criteria	Hit threshold = Average "% stabilization" of all test samples (0,24%) + 3 times standard deviation of all test samples (3*3,99%) = 11.7%
	Hit rate	0.6%
	Additional assay(s)	Retesting of hits in CETSA screening assay at 11 concentrations.<br>Retesting of a selection of hits in screening assay at 11 concentrations as a function of incubation time.<br>Selected hits tested using Western blot based CETSA protocol.<br>Measurements of intracellular concentrations for selected hits.<br>Selected compound verified using Tm shifts, surface plasmon resonance and co-crystallization.
	Confirmation of hit purity and structure	ID and purity analysis with LC-UV/MS detection
	Additional comments	

Supplementary Table 3| Summary data for identified hits in the CETSA-HTS on thymidylate synthase

Compound ID	Structure	Listed drug in THOMSON REUTERS Integrity	Name	% stabilization in screen	Hit confirmation ITDRF $_{CETSA}$	New dilutions ITDRF $_{CETSA}$	ITDRF $_{CETSA}$	Expected mass found	% Purity
**Nucleosides**									
CBK041650	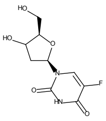	Yes	Floxuridine<br>5-fluorodeoxyuridine	64	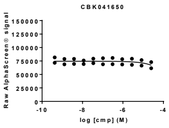	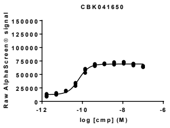	6.61E-11	YES	N.D.
CBK201031	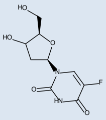	Yes	Floxuridine<br>5-fluorodeoxyuridine	66	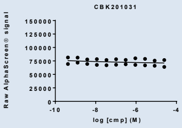			N.D.	N.D.
CBK011695	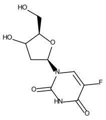	Yes	Floxuridine<br>5-fluorodeoxyuridine	56	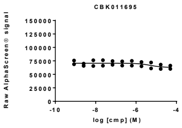			YES	100
CBK200925	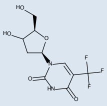	Yes	Trifluridine<br>Trifluorothymidine	66	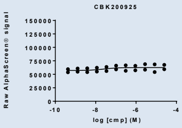	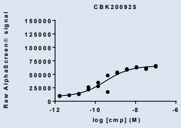	3.18E-10	N.D.	N.D.
CBK201207	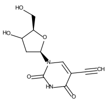			102	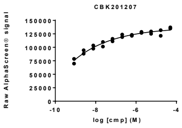	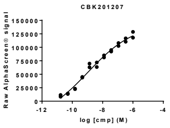	1.41E-09	YES	100
CBK201329	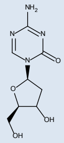	Yes	Decitabine<br>5-Aza-deoxycytidine	91	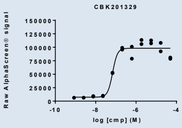		6.87E-08	YES	100
CBK201230	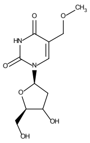			23	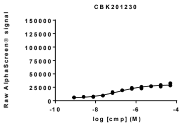		1.42E-07	YES	66
CBK201221	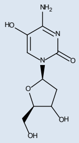			22	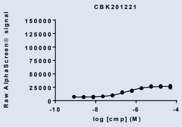		3.43E-07	YES	100
CBK201184	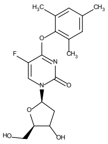			66	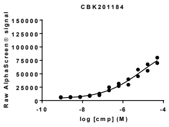		9.17E-06	YES	100
CBK201182	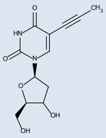			90	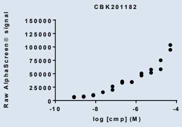		2.28E-05	YES	100
CBK201253	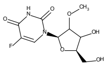			61	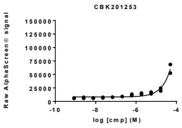		N.D.	YES	100

CBK011636	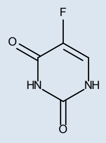	Yes	5-Fluorouracil Fluorouracil	48	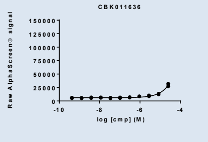		N.D.	N.D.	N.D.
CBK200768	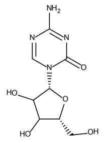	Yes	Azacitidine 5-Azacytidine	32	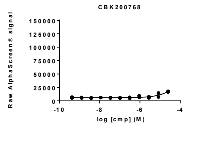		N.D.	YES	100
CBK201216	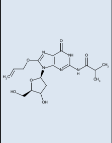			30	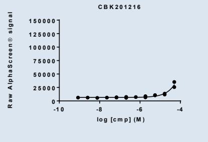		N.D.	YES	100
CBK201267	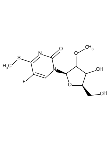			23	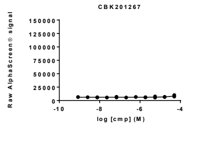		N.D.	YES	100
CBK201195	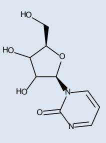			22	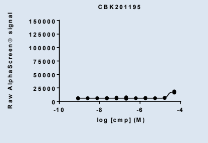		N.D.	YES	100
CBK201302	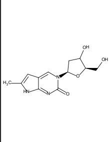			21	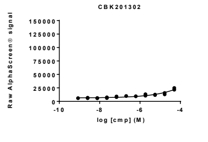		N.D.	YES	100
CBK015742	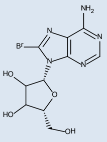			20	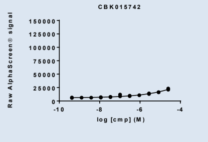		N.D.	YES	100
CBK201179	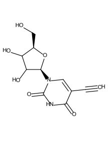			20	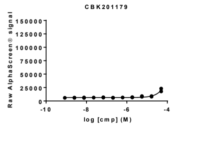		N.D.	YES	100
CBK201287	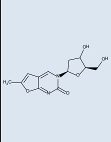			20	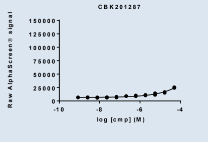		N.D.	YES	100
CBK201174	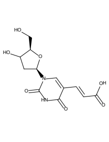			17	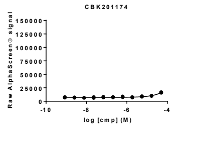		N.D.	YES	100
CBK201239	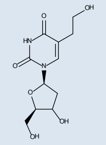			17	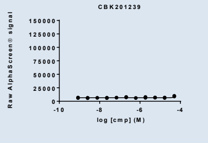		N.D.	YES	100
CBK201249	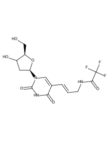			17	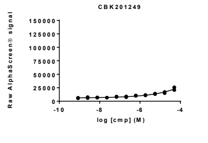		N.D.	YES	99

CBK201265	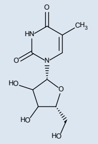			17	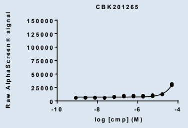		N.D.	YES	100
CBK201188	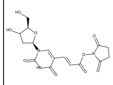			16	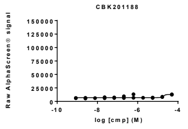		N.D.	YES	92
CBK201262	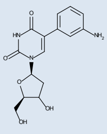			16	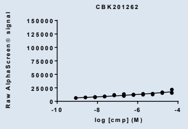		N.D.	NO	100
CBK201317	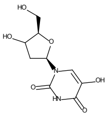			16	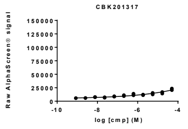		N.D.	YES	100
CBK201310	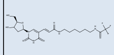			15	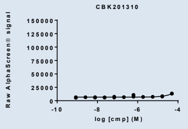		N.D.	YES	93
CBK201330	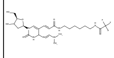			13	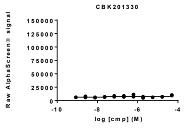		N.D.	YES	95
**Anti-folates and similar structures**									
CBK200695	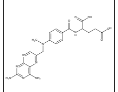	Yes	Amethopterin	94	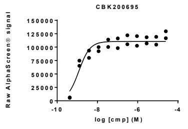		9.42E-10	YES	90
CBK041938	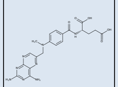	Yes	Methotrexate	96	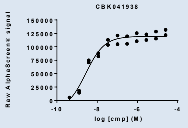		2.70E-09	YES	98
CBK041886	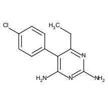	Yes	Pyrimethamine	76	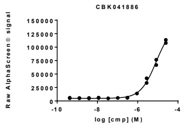		8.38E-06	YES	99
CBK012158	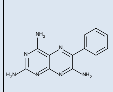	Yes	Triamterene	78	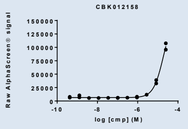		N.D.	YES	99

Other									
CBK200591N	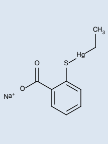	Yes	Thimerosal<br>Thiomersal	115	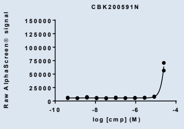		N.D.	YES	100
CBK200926	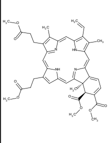			81	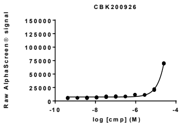		N.D.	YES	99
CBK028412T	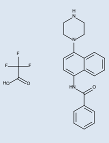			64	Out of stock		N.D.	N.D.	N.D.
CBK061572	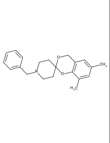			28	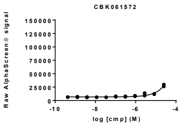		N.D.	YES	100
CBK041969	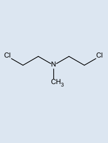	Yes		24	Out of stock		N.D.	N.D.	N.D.
CBK200931G	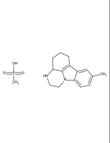	Yes	Pirlindole<br>Pyrazidol	23	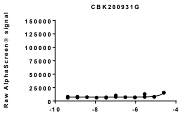		N.D.	YES	99
CBK126784	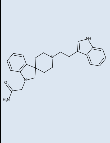			22	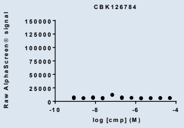		N.D.	YES	96
CBK041519C	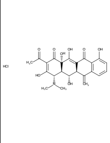			20	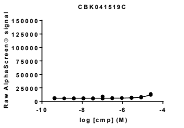		N.D.	NO	N.D.
CBK115334	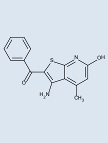			20	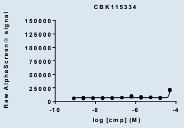		N.D.	YES	100
CBK106797	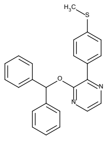			19	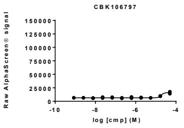		N.D.	YES	80
CBK088843	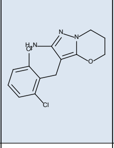			18	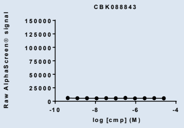		N.D.	YES	100
CBK042304	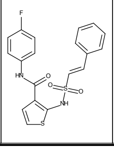			17	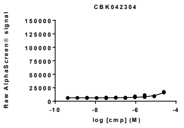		N.D.	YES	90

CBK041124C	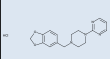	Yes	Piribedil	17	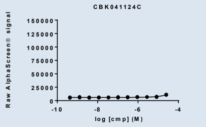		N.D.	NO	100
CBK042297	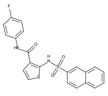			16	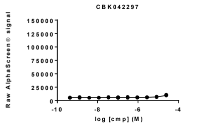		N.D.	YES	95
CBK012353C	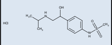	Yes	Sotalol	14	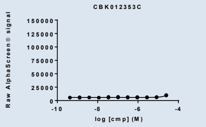		N.D.	YES	100
CBK042169C	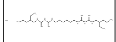			14	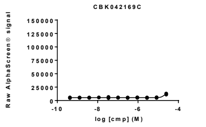		N.D.	YES	95
CBK023984C	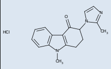			13	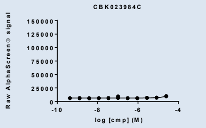		N.D.	YES	100
**Inactive in hit confirmation**									
CBK088089C	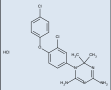			63	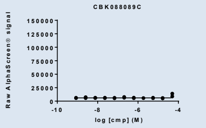		N.D.	YES	98
CBK134216	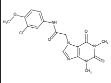			50	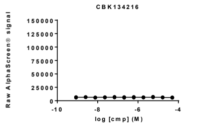		N.D.	YES	100
CBK087417	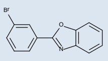			76	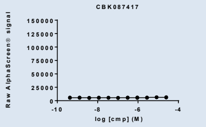		N.D.	YES	92
CBK201030	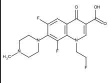	Yes	Fleroxacin	66	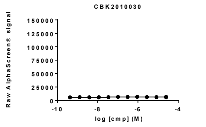		N.D.	YES	100
CBK200917G	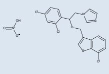	Yes	Sertaconazole nitrate	66	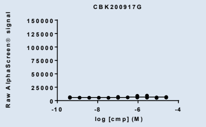		N.D.	YES	100
CBK041793	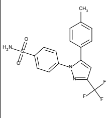	Yes	Celecoxib	61	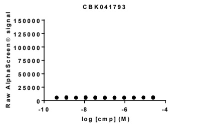		N.D.	YES	100

CBK041301C	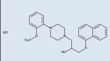	Yes	Naftopidil	59	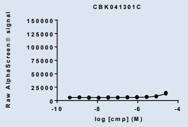		N.D.	YES	99
CBK200886B	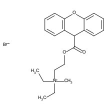			58	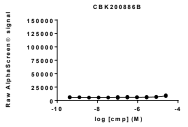		N.D.	YES	95
CBK200094	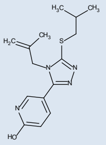			58	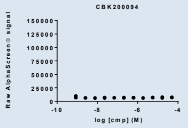		N.D.	YES	100
CBK200950	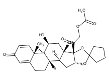	Yes	Amcinonid<br>Amciderm<br>Cyclocort<br>Penticort<br>Visderm	56	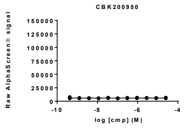		N.D.	YES	100
CBK087111	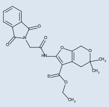			55	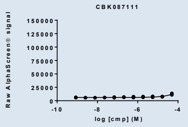		N.D.	YES	95
CBK006628	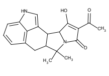			55	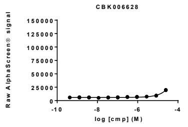		N.D.	YES	84
CBK087105	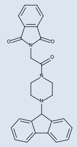			52	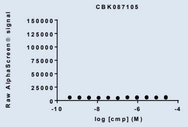		N.D.	YES	93
CBK015160	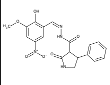			52	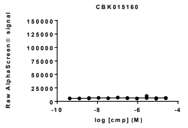		N.D.	YES	93
CBK060013	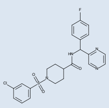			34	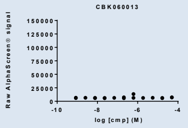		N.D.	YES	87

Supplementary Table 4| Shortlist of compounds that were investigated as potential actives based on structural resemblance to known thymidylate synthase inhibitors

Compound ID	Structure	% stabilization in screen
**Uracil-based analogues**		
CBK201207	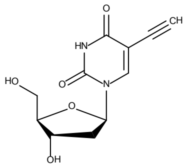	102
CBK201182	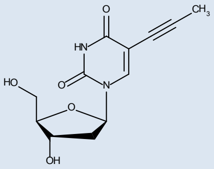	90
CBK201031	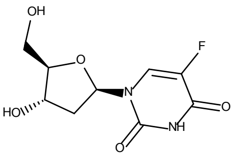	66
CBK200925	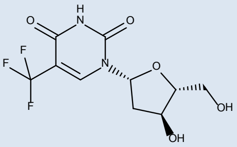	66
CBK201184	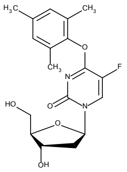	66
CBK041650	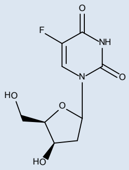	64
CBK201253	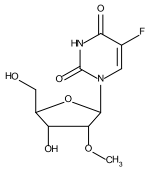	61
CBK011695	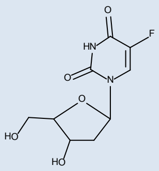	56

CBK201230	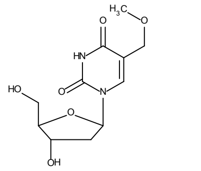	23
CBK201179	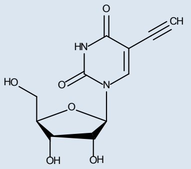	20
CBK201287	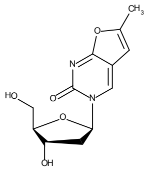	20
CBK201174	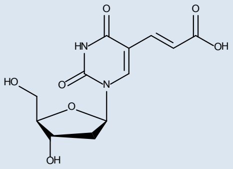	17
CBK201239	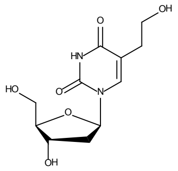	17
CBK201249	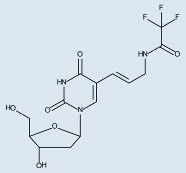	17
CBK201265	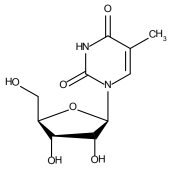	17
CBK201188	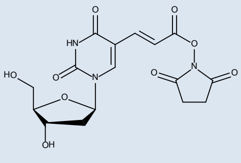	16
CBK201262	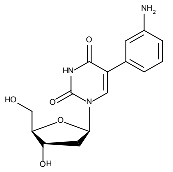	16
CBK201317	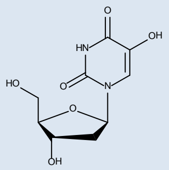	16

CBK201310	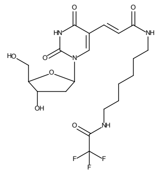	15
CBK200650	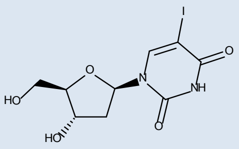	10
CBK201214	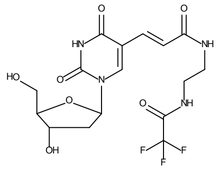	9
CBK201332	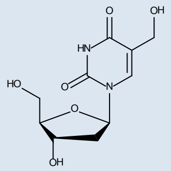	9
CBK200650	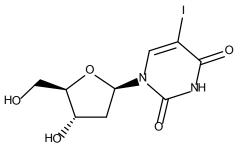	8
CBK201278	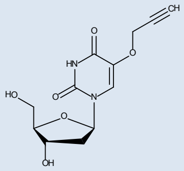	7
CBK201211	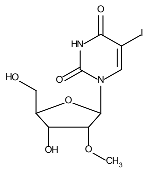	5
CBK201338	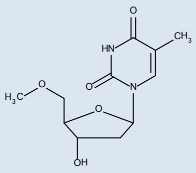	4
CBK041677	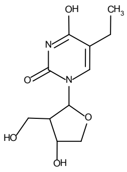	3
CBK201166	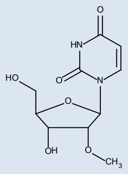	1

CBK087642	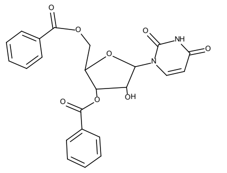	0
CBK201159	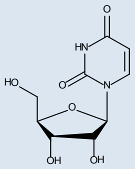	0
CBK201200	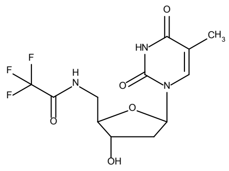	0
CBK201279	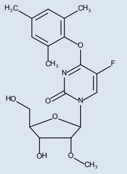	0
CBK201256	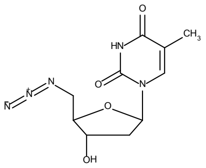	0
CBK201280	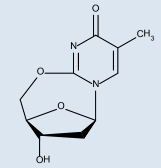	0
CBK201180	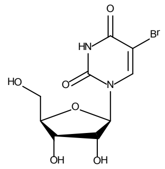	-1
CBK201339	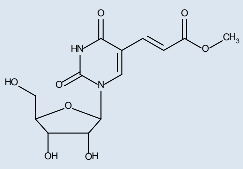	-1
CBK201270	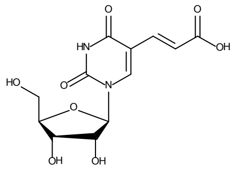	-1
CBK201316	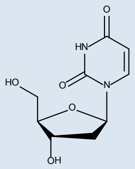	-1

CBK201304	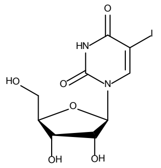	-1
CBK011998	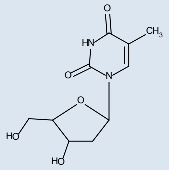	-1
CBK041618	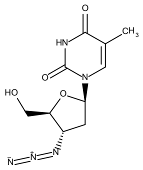	-1
CBK201298	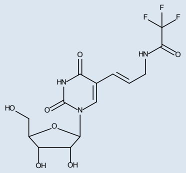	-1
CBK201334	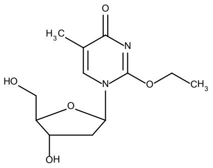	-1
CBK201315	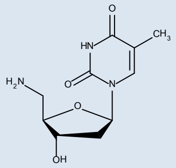	-1
CBK201232	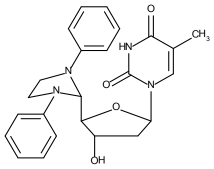	-1
CBK201281	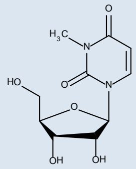	-1
CBK201231	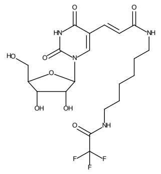	-1
CBK201251	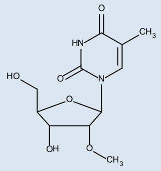	-1

CBK201322	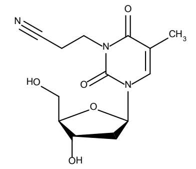	-1
CBK201197	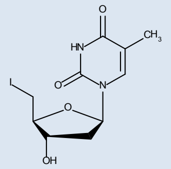	-1
CBK201283	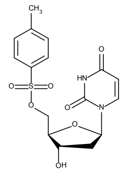	-2
**Cytosine-based analogues**		
CBK201221	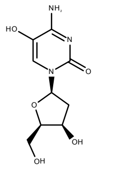	22
CBK201195	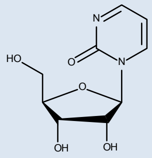	22
CBK201302	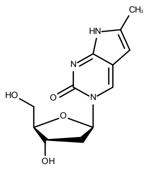	21
CBK201330	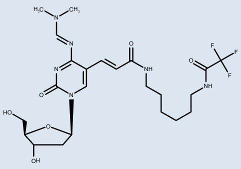	13
CBK201237	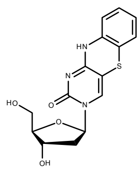	9
CBK201226	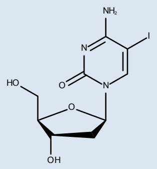	7

CBK201161	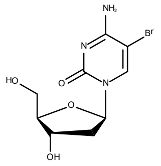	5
CBK201307	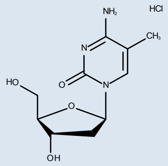	4
CBK200928	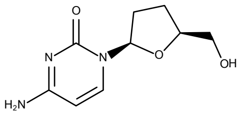	0
CBK201311	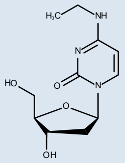	0
CBK201268	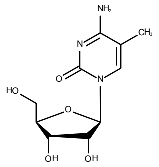	0
CBK201235	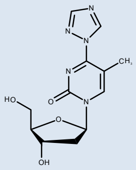	0
CBK201227	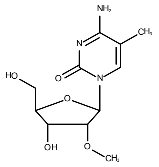	0
CBK200978	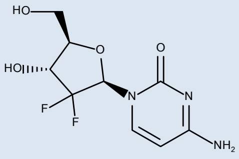	0
CBK201203	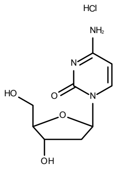	0
CBK201296	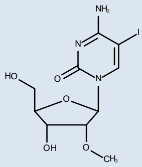	0

CBK201274	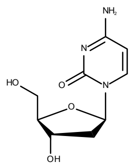	-1
CBK201219	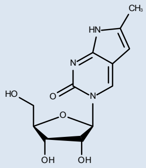	-1
CBK042058	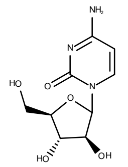	-1
CBK200603	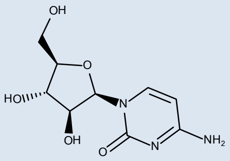	-1
CBK024684	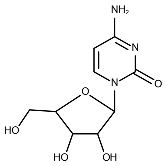	-1
CBK201306	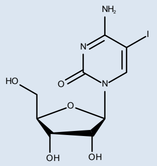	-1

These compounds were identified based on sub-structure searches within the primary screening set of 10,928 compounds. These searches were based on uridine, cytidine and anti-folate like compounds according to the following rules:

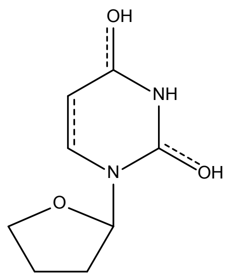

a

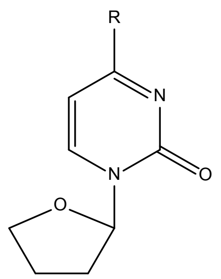

b

R= N or H

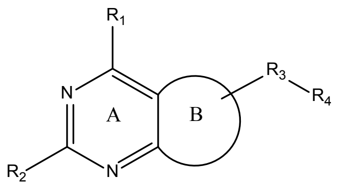

c

Ring A
No substitution on N allowed
R_1 or R_2 may not be H
RingB
5 or 6 membered ring
0-2 N i the ring the rest=C,
R_3 is 0-2 atoms
R_4 is an aromatic ring

The pyrimidine rings in (**a**) and (**b**) were allowed to vary in regards to any substitutions on the furan ring and on the base and included also variations of the bond character, *i.e.* single/double bonds as indicated in the structures. The general minimal structure element for anti-folate like structures (**c**) was determined based on a series of known inhibitors that has been tested in man as drugs or putative drugs targeting thymidylate synthase. Based on these criteria the searches resulted in 51 uracil-based analogs, 22 cytosine-based analogs and no anti-folate like structures in the screening library.

Supplementary Table 5| Additional data for compounds that were investigated as potential false negatives based on structural resemblance to known thymidylate synthase inhibitors

Compound ID	Structure	% stabilization at screen (2h preincubation)	% stabilization at retest (6h preincubation)
CBK200978	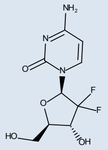	0	3
CBK201214	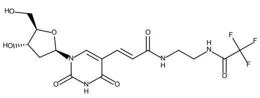	9	6
CBK200650	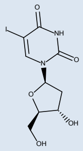	10	14
CBK201332	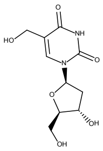	9	7
CBK201168	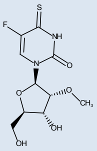	2	5
CBK201278	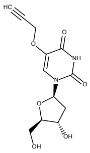	7	12

To establish whether there were any obvious false negatives missed in the screen we used substructure searches based on clinically used TS inhibitors (see Methods for details and **Supplementary Table 4** for the outcome). Manual curation revealed the presence of these six compounds that motivated further experimental examination, including the cytidine analog gemcitabine, which was recently shown to weakly bind TS following phosphorylation and deamination to the corresponding uridine analog[5]. None of these resulted in significant target engagement using the present assay setup, even after prolonged pre-incubation times, thus confirming the original screen data. Apart from gemcitabine, these compounds are not previously reported as TS inhibitors showing that the CETSA-HTS approach selected accurately for known inhibitors with a low rate of false negatives.

Supplementary Table 6| Historical screening data for CBK115334

Target source	Assay type	Detection type	Concentration (M)	Inhibition (%)	Activation (%)	Hit
Embryonic stem cells	Cell-based	Luminescence	1,0E-05		-12,5	No
Enzyme membrane preparation	Enzymatic	Absorbance	1,0E-05	12,2		No
Recombinant enzyme	Enzymatic	Absorbance	1,0E-05	25,6		No
Recombinant enzyme	Enzymatic	Absorbance	1,0E-05	18,3		No
Recombinant enzyme	Enzymatic	Absorbance	1,0E-05	12,7		No
Recombinant enzyme	Enzymatic	Absorbance	1,0E-05	2,3		No
Recombinant enzyme	Enzymatic	Absorbance	1,0E-05	-3,9		No
Recombinant enzyme	Enzymatic	Absorbance	1,0E-05	-2,5		No
Mammalian cell-line	Cell-based	Imaging	1,3E-05	-28,3		No
Mammalian cell-line	Cell-based	Luminescence	1,0E-05		27,2	No
Mammalian cell-line	Cell-based	Imaging	1,0E-05	-3,9		No
Recombinant enzyme	Binding	Fluorescence	1,0E-05		4,7	No
Recombinant enzyme	Enzymatic	Absorbance	1,0E-05	-0,1		No
Recombinant enzyme	Enzymatic	Fluorescence	1,0E-05	-11,0		No
Recombinant enzyme	Enzymatic	Fluorescence	1,0E-05	1,2		No
Recombinant enzyme	Enzymatic	Absorbance	5,0E-06	-2,3		No
CETSA in mammalian cell-line	Cell-based	Luminescence	5,0E-05		19,5	Yes
Recombinant enzyme	Binding	Luminescence	1,5E-05	1,0		No
Recombinant enzyme	Binding	Luminescence	1,0E-05	12,3		No

Supplementary Note 1| Abbreviations

CBCS	Chemical Biology Consortium Sweden
CDA	Cytidine deaminase
CETSA	Cellular thermal shift assay
DCK	Deoxycytidine kinase
DCTD	Deoxycytidylate deaminase
DMSO	Dimethyl sulfoxide
dUMP	2´-deoxyuridine 5´-monophosphate
EdU	5-ethynyl-2´-deoxyuridine
EdUMP	5-ethynyl-2´-deoxyuridine 5´-monophosphate
FdU	5-fluoro-2´-deoxyuridine; floxuridine
FdUMP	5-fluoro-2´-deoxyuridine 5´-monophosphate
5-FU	5-fluorouracil
FUR	5-fluorouridine
HTS	High-throughput screening
$ITDRF_{CETSA}$	Isothermal dose-response fingerprint
LC-MS/MS	Liquid chromatography-tandem mass spectrometry
S.E.M.	Standard error of mean
SPR	Surface plasmon resonance

T_{agg}	Aggregation temperature
TFT	5-trifluoro-2´-deoxythymidine
TFTMP	5-trifluoro-2´-deoxythymidine 5´-monophosphate
TS	Thymidylate synthase

Supplementary References

1. Fisher, T. C. *et al.* bcl-2 modulation of apoptosis induced by anticancer drugs: Resistance to thymidylate stress is independent of classical resistance pathways. *Cancer Res.* **53,** 3321–3326 (1993).

2. Jafari, R. *et al.* The cellular thermal shift assay for evaluating drug target interactions in cells. *Nat. Protoc.* **9,** 2100–2122 (2014).

3. Litvinov, V. P. *et al.* New method for the synthesis of substituted 2-pyridones. *Izv. Akad. Nauk SSSR, Ser. Khim.* 1869–1870 (1984).

4. Dyachenko, V. D. *et al.* Cyclization reactions of nitriles. XXXIX. Synthesis and transformations of 6-hydroxy-3-cyano-2(1H)-pyridinechalcogenones. *Zh. Obs. Khim.* **60,** 2384–2392 (1990).

5. Honeywell, R. J., Ruiz van Haperen, V. W. T., Veerman, G., Smid, K. & Peters, G. J. Inhibition of thymidylate synthase by 2’,2'-difluoro-2'-deoxycytidine (Gemcitabine) and its metabolite 2',2'-difluoro-2'-deoxyuridine. *Int. J. Biochem. Cell Biol.* **60,** 73–81 (2015).
